# Transcriptomic insights into arabinogalactan protein mechanism of action in galactosyltransferase octuple mutants

**DOI:** 10.3389/fpls.2025.1706954

**Published:** 2026-01-16

**Authors:** Damilola A. Ayorinde, Gbolaga O. Olanrewaju, Allan M. Showalter

**Affiliations:** 1 Molecular and Cellular Biology Program, Athens, OH, United States; 2 Department of Environmental and Plant Biology, Athens, OH, United States; 3 Biomedical Sciences, Ohio University, Athens, OH, United States

**Keywords:** arabinogalactan proteins, cell signaling, cell wall, development, glycosylation, reproduction, stress response

## Abstract

Arabinogalactan-proteins (AGPs) are a family of hyperglycosylated hydroxyproline-rich glycoproteins essential for plant growth and development and generally contain 10% protein and 90% carbohydrate. Eight galactosyltransferases (GALTs), specifically GALT2-GALT9, catalyze galactose addition to hydroxyproline residues in the AGP protein backbone and initiate glycosylation of AGPs. Arabidopsis *galt* octuple mutants that result from the knockout of eight *GALT* genes displayed severe phenotypic changes, prompting our exploration of the mechanisms of action of AGPs by comparing the transcripts of *galt* octuple mutant flowers and siliques to wild type flowers and siliques in Arabidopsis thaliana. Transcriptomic analysis of flowers from *galt* octuple mutants revealed 930 significantly differentially expressed genes (426 upregulated, 504 downregulated). Many of the downregulated genes are reported to be crucial for pollen tube growth, pollination, and flower development. In siliques, there were 1,476 significantly differentially expressed genes (1,027 upregulated, 449 downregulated), including the downregulation of genes for pectin methyl esterase inhibitors (PMEIs) and suspensor development. There were 45 genes commonly downregulated in flowers and siliques, which are reportedly crucial for glycosylation, glycoprotein synthesis, and cell wall modification. On the other hand, there were 194 commonly upregulated genes linked to calcium ion binding with kinases and phosphatases in the signal transduction pathways, cell-cell communication, stress response and pathogen defense response regulation in both flowers and siliques. These findings offer insights into plant molecular responses to AGP dynamics and provide a foundation for further investigations into the underlying mechanisms of action of AGPs by revealing the genes and pathways related to AGP function, suggesting that AGPs may mediate the effects of these genes or pathways, in part, by influencing signal transduction pathways involving kinases and phosphatases.

## Introduction

Arabinogalactan-proteins (AGPs) are hydroxyproline-rich glycoproteins that move through the secretory pathway to the plasma membranes and cell walls of various plant tissues and organs (Seifert and Roberts, 2007; Silva et al., 2020). They play major roles in plant development, signaling pathways, and cell wall integrity (Ma et al., 2022; Seifert, 2021). AGPs are implicated in the regulation of root growth (Bossy et al., 2009; Dolan et al., 1995), stem development, and differentiation (Ito et al., 2005; Liu et al., 2020; MacMillan et al., 2010), cell expansion and division, embryogenesis of somatic cells (Pérez-Pérez et al., 2019), sexual reproduction (Nguema-Ona et al., 2012; Pereira et al., 2015; Su and Higashiyama, 2018), fruit ripening (Leszczuk et al., 2020), wound healing, response to abiotic stress factors, and interaction with microorganisms (Mareri et al., 2019; Nguema-Ona et al., 2013; Rashid, 2016; Seifert, 2021). AGPs also interact with other cell wall polymers, specifically pectin and hemicellulose, to form a complex network that is important in regulating the cell wall dynamics (Velasquez et al., 2011).

In Arabidopsis, over 80 AGPs have been identified (Showalter, 2010), and they have a structure which has a protein backbone that is glycosylated with galactose, arabinose, glucuronic acid and rhamnose. The protein backbone is hydroxyproline-rich, and contains other abundant amino acids including alanine, serine and threonine and often contains in repeated dipeptide sequences such as Ala-Hyp and Ser-Hyp. The carbohydrate moiety makes up ~90% of a typical AGP, while the protein backbone makes up the remaining ~10%. The carbohydrate moiety plays a major role in the function of AGPs, as it constitutes the interactive molecular surface for AGPs to bind to other molecules. Glycosylation also creates patterns that can be recognized by signaling molecules such as receptor-like kinases (Borassi et al., 2020).

AGP glycosylation is a post-translational modification that occurs in the Golgi apparatus by the sequential action of enzymes called glycosyltransferases (GTs), including the GT 31 family of glycosyltransferases (Keegstra, 2010). The polypeptide component of glycoproteins is generated first, followed by carbohydrate side chains during passage through the endoplasmic reticulum and Golgi apparatus. Glycosyltransferases are the builders of AGP glycan chains, and of these glycosyltransferases, eight galactosyltransferases from the GT 31 family have been found to initiate glycosylation of AGPs by the addition of the first sugar, galactose. These enzymes were named GALT 2-GALT9 (Basu et al., 2015; Dilokpimol et al., 2014; Ogawa-Ohnishi and Matsubayashi, 2015; Qu et al., 2008). Plants lacking one or two of these enzymes display various abnormal, albeit subtle phenotypes (Basu et al., 2015; Zhang et al., 2021). However, due to redundancy within this family of enzymes, the other family members compensate for the one or two that are mutated (Zhang et al., 2021). Therefore, to understand the functions of these enzymes, all eight genes encoding these enzymes were ultimately mutated/knocked-out, creating an *galt* octuple mutant (Kaur et al., 2023). The *galt2 galt5* double mutant and *hpgt* triple mutant have been shown to have ~ 40% and ~ 70% reductions in glycosylated AGPs in young seedlings, respectively (Basu et al., 2015). Higher-order mutants lacking five GALTs (*galt2 galt3 galt4 galt5 galt6*) show drastically reduced AGP glycosylation, with lower β-Gal-Yariv-precipitated AGPs and reduced arabinose/galactose in rosette leaves, stems, and siliques compared to wild-type (Basu et al., 2015; Zhang et al., 2021). Specifically, the octuple mutant displays not only a significant (~50%) reduction in the amount of Yariv precipitable AGPs in both flowers and siliques, but also a significant (~60%) reduction in the number of Hyp-arabinogalactan chains present in the Yariv precipitable AGPs in both flowers and siliques (Kaur et al., 2023; Moreira et al., 2023). Moreover, flower and silique development in the octuple mutant is significantly impaired compared to wild type plants based on comparative morphological and microscopic time course observations (Kaur et al., 2023). The octuple mutants displayed severe abnormal phenotypes including but not limited to a delay in bolting, underdeveloped anthers that affects fertilization, shorter siliques, and fewer seeds (Kaur et al., 2023; Moreira et al., 2023, 2024).

Although considerable research has been carried out on AGPs to understand their structure and functions, their mechanism of action remains a mystery. To understand the mechanism of action of AGPs, we decided not to focus on one AGP but to leverage on the fact that glycosylation is a crucial part of AGP biosynthesis. Glycosylation is essential for the functions of classical AGPs, where type II arabinogalactan polysaccharides dominate biological activity. In non-classical AGPs, glycosylation remains important but often acts in concert with non-AGP domains in the protein to mediate function (Leszczuk et al., 2023). With the understanding that the process of glycosylation and synthesis of AGPs was disrupted in the generated *galt* octuple mutants, we decided to study the mechanism of action of AGPs through these mutants. Considering the abnormal phenotypes such as abnormal phenotypes of delayed bolting, underdeveloped anthers, shorter siliques and fewer seeds observed in the *galt* octuple mutant, we found that the flowers and siliques have the most obvious phenotypic abnormalities. Using publicly available databases such as TAIR (www.arabidopsis.org), ePlant (bar.utoronto.ca/eplant/) and Arabidopsis Developmental Atlas Viewer (http://arabidopsisdevatlas.salk.edu/), we explored the expression patterns of the eight *GALT* genes in flowers and siliques. These genes show coherent expression during silique and flower development. Additionally, other studies have shown the expression of these *GALT* genes in flowers or siliques (Basu et al., 2015, 2016; Ogawa-Ohnishi and Matsubayashi, 2015).

In this study, we compared the transcripts of the *galt* octuple mutants to wild-type Arabidopsis plants using transcriptomic analysis/RNA-seq. RNA-seq is a method that used to study gene expression at the transcript level and reveals genes that are differentially expressed as a result of a treatment or condition, as well as the genes and metabolic pathways that are connected to the expression of our desired genes. Thus, in this study, we used RNA-seq to compare gene expression in *galt* octuple mutant flowers and siliques with wild-type controls, identifying differentially expressed genes (DEGs) and associated pathways. Consequently, this work provides insight to the genes involved and associated with the mechanism of action of AGPs and to the various molecular and biological processes connected to AGPs, particularly in reproductive tissues.

## Materials and methods

### RNA extraction

The upper part of the inflorescence of mature flowers (stage 12-14, Smyth et al., 1990) were sampled at 35 DAG (wild type) and 45 DAG (mutant) from the primary inflorescence. Siliques were harvested at 10 days after pollination, which is 45 DAG (wild type) and 55 DAG (mutants), ensuring developmental equivalence due to the mutant’s known developmental delay. We selected 10 DAP siliques because we wanted a stage where the siliques are fully developed and the seeds are in active development prior to senescence. The organs were frozen in liquid nitrogen and subsequently ground into a powder with a mortar and pestle before RNA extraction. Total RNA was extracted from the ground tissues using a QIAGEN RNeasy^®^ Plant Mini Kit (QIAGEN, Cat. No. 74903). The samples, which included four replicates each of the *galt* octuple mutant and wild type flowers and siliques, were stored at -80°C. RNA integrity was assessed using a NanoDrop 2000 spectrophotometer and an Agilent Bioanalyzer. Samples with RIN scores >7 were shipped on dry ice to Azenta Life Science Facility for sequencing using Illumina Hiseq, obtaining paired-end (PE) reads with an average length of 150 bp, ~350M PE reads (~105GB), single index per lane.

### cDNA library construction and Illumina sequencing

Libraries were first constructed using the Illumina Stranded mRNA TruSeq kit, and the libraries were sequenced. A quality check was conducted on the raw data. The reads were then mapped to a reference genome.

### Read alignment and read count

Sequence reads were trimmed using Trimmomatic v0.36 to remove adapter sequences and low-quality nucleotides. The resulting trimmed reads were aligned to the Arabidopsis thaliana ENSEMBL TAIR10 reference genome from ENSEMBL using STAR aligner v2.5.2b, a splice-aware aligner that identifies and incorporates splice junctions to improve read alignment. This process generated Binary Alignment Map (BAM) files. Unique gene hit counts were obtained using feature Counts from the Subread package v1.5.2. Counts were summarized based on the transcript_id feature in the annotation file, considering only unique reads mapping to exon regions. Strand-specific counting was applied if the library preparation was strand-specific.

### Differentially expressed gene analysis

Following gene hit count extraction, the resulting read count table was used for downstream differential expression analysis. Expression profiling was subsequently performed using DESeq2. DESeq2 was employed to compare gene expression between user-defined sample groups, with the Wald test generating p-values and log2 fold changes, using the R studio package. Genes were classified as differentially expressed if they had an adjusted p-value (padj) < 0.05 and an absolute log2 fold change (Log2FC) > 1. DESeq2 data was also used for the gene ontology and functional annotation.

### Gene ontology and enrichment analysis

Gene ontology analysis was performed on the statistically significant set of genes by implementing the software ShinyGO v0.82 and iDEP 8.0. The TAIR GO list was used to cluster the set of genes based on their biological processes and determine their statistical significance. A list of genes clustered based on their gene ontologies was generated.

## Results

### Identification and analysis of differentially expressed genes

**Figure 1:**
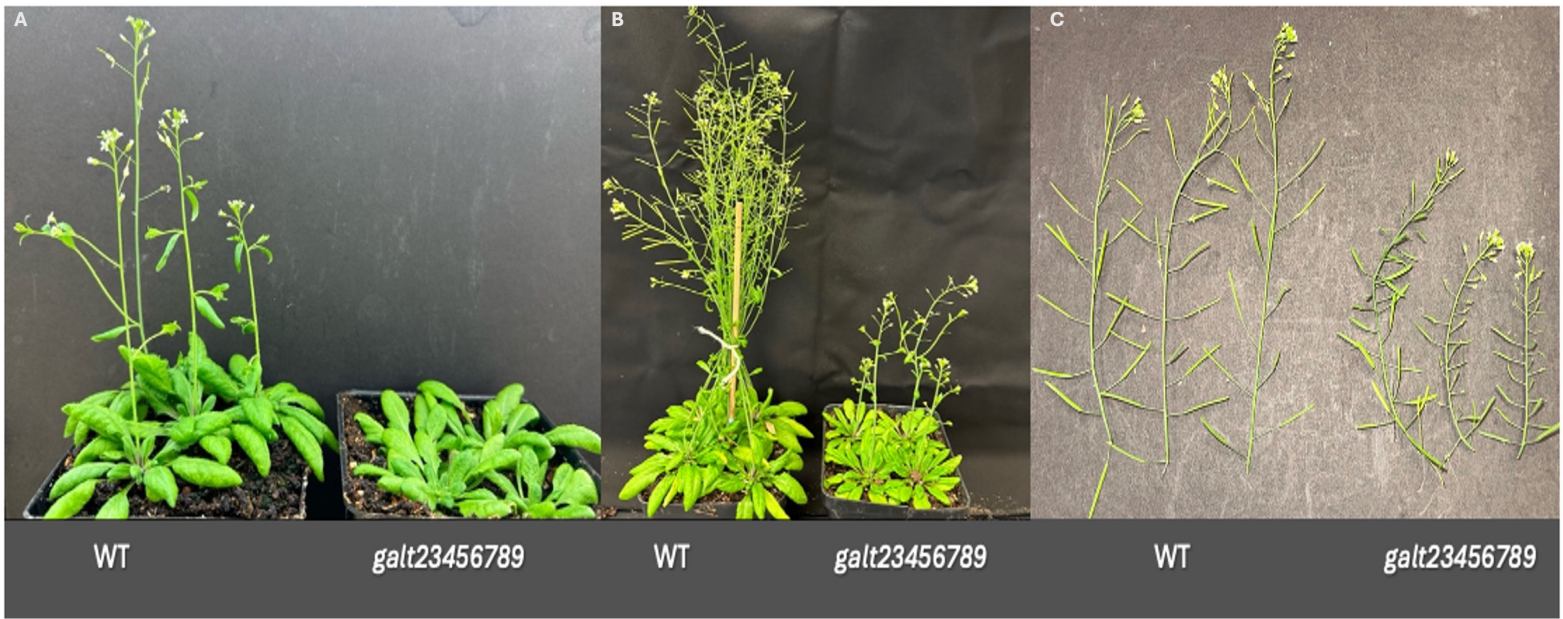
Phenotypic differences displayed by wild-type Arabidopsis and galt octuple mutant plants. **(A)** Wild-type Arabidopsis and galt octuple mutant plants at 35 days after germination (DAG). **(B)** Wild-type Arabidopsis and galt octuple mutant plants at 45 DAG. **(C)** Wild-type Arabidopsis and galt octuple mutant inflorescences and siliques at 45 DAG. The galt octuple mutant, also named galt23456789, exhibited delayed bolting, underdeveloped flowers, and shorter siliques compared to the wild-type plants, reflecting severe developmental abnormalities resulting from the loss of eight galactosyltransferase (GALT) genes responsible for AGP glycosylation.

**Figure 2:**
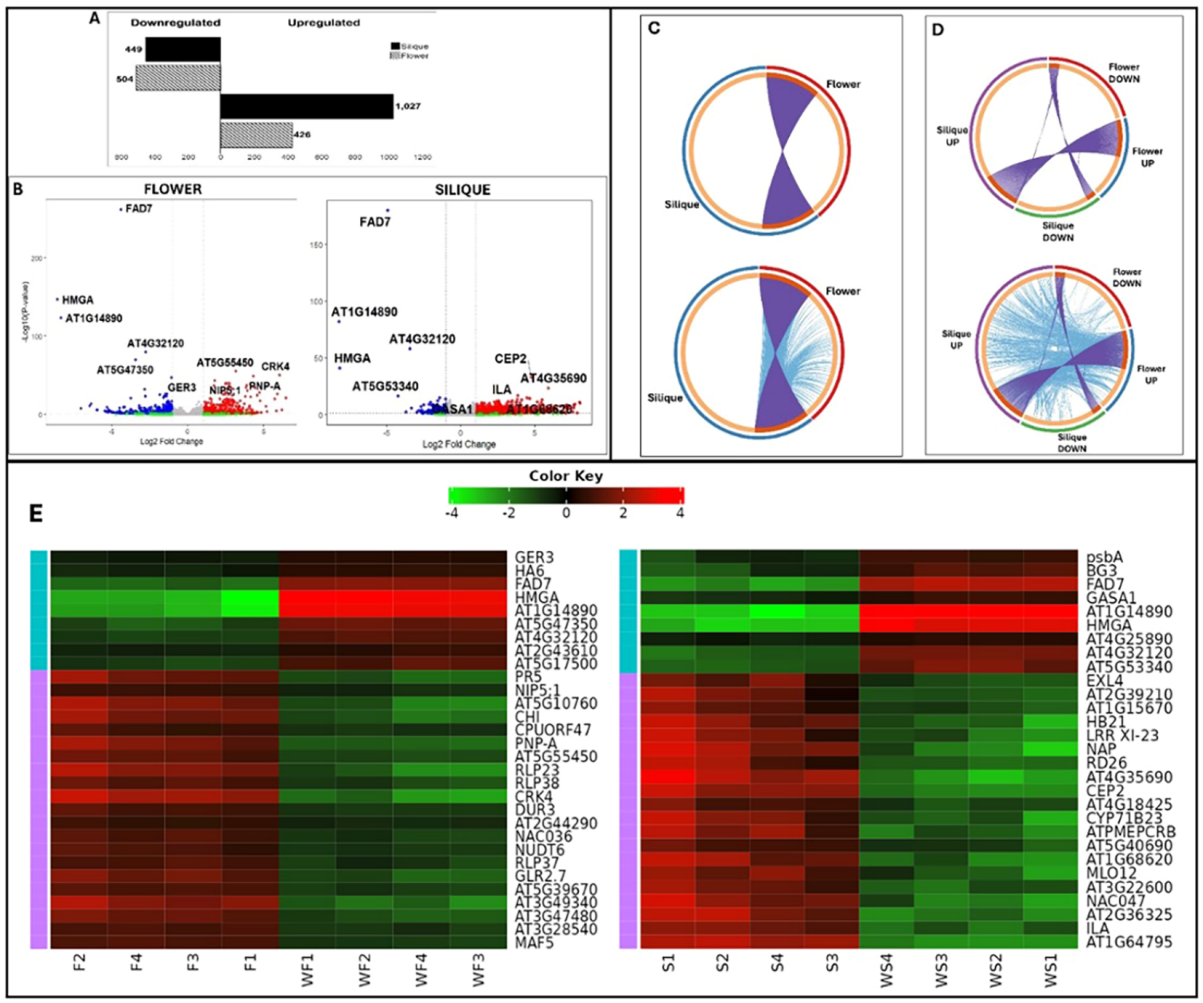
Comparative transcriptomic analysis of *galt* octuple mutant and wild-type Arabidopsis flowers and siliques. **(A)** Bar plot summarizing the number of significantly upregulated and downregulated genes in flowers and siliques of *galt* octuple mutants relative to wild-type plants. Siliques exhibited a markedly higher number of upregulated genes compared to flowers, indicating stronger transcriptional reprogramming. **(B)** Volcano plots showing differentially expressed genes (DEGs) in *galt* octuple mutant versus wild-type flowers (left) and siliques (right). Labeled genes represent the top statistically significant expressed genes. Blue-marked genes are downregulated (Log2fold change < -1). Red-marked genes are upregulated (Log2fold change > 1). Green-marked genes are non-statistically significant (*p>0.05*), but have Log2fold change above the threshold, while grey-marked genes are neither significant nor meet the Log2fold change threshold. There is nothing like 3/4 in there. **(C)** Chord diagrams depicting overlaps in DEGs (top) and enriched functional pathways (bottom) between flower and silique datasets, regardless of regulatory direction. **(D)** Directional chord diagrams separating upregulated and downregulated genes (top) and functional pathways (bottom) across flowers and siliques. **(E)** Heatmap of top differentially expressed genes (DEGs) in galt octuple mutant flowers and siliques. The heatmap displays the log2 fold changes of the top 20 DEGs (based on magnitude and adjusted p-value) across both tissues, with upregulation shown in red and downregulation in blue.

Seeds from the *galt* octuple mutants and wild-type Arabidopsis plants were planted to observe and confirm the reported phenotypic abnormalities in *galt* octuple mutants (**Figure 1**) (Kaur et al., 2023; Moreira et al., 2024). To investigate the transcriptional consequences of impaired arabinogalactan-protein (AGP) galactosylation, we chose to compare the transcriptomes of flowers and siliques from wild-type and *galt* octuple mutant Arabidopsis plants. As an initial validation of the RNA-seq data and the octuple mutant, we confirmed that transcripts for all eight targeted *GALT* genes (*GALT2-9*) were significantly downregulated in both flowers and siliques compared to wild-type. The reduction of AGP glycosylation due to the knockout of galactosyltransferases in the *galt* octuple mutant resulted in widespread transcriptomic changes in both flowers and siliques, but these changes varied significantly between the two organs. A total of 930 genes were differentially expressed in flowers, whereas 1,476 were differentially expressed in siliques, witha higher number of genes upregulated in the *galt* octuple mutant in siliques (1,027) compared to flowers (426) (**Figure 2A**). The number of downregulated genes was not so different between organs, with 449 genes downregulated in siliques and 504 genes downregulated in flowers (**Figure 2A**). This suggests a greater compensatory or stress-induced transcriptional response in siliques, which may reflect their involvement in seed development and their higher reliance on intact cell wall architecture and signaling integrity during post-fertilization growth (Di Marzo et al., 2022; Doughty et al., 2014; Herrera-Ubaldo and De Folter, 2022). Volcano plots (**Figure 2B**) were used to display the distribution of gene expression changes, with selected top genes labeled based on their statistical significance. Some genes, such as *FAD7, HMGA, AT1G14890*, and *AT4G32120*, were highly downregulated in both tissues. Others, like *CEP2, ILA, and AT4G35690*, were specifically upregulated in siliques while *AT5G55450, CRK4, PNP-A and NIP5–1* were specifically upregulated in flowers. Chord plots in **Figure 2C** show global overlap and divergence between flower and silique expression profiles (**Figure 2C**). **Figure 2D** further distinguishes the relationships among upregulated and downregulated genes across tissues. The arcs represent genes shared between tissues, with ribbons showing the direction and extent of overlap. While some genes were co-regulated, others displayed opposite trends between flowers and siliques. Analysis of the overlap between DEGs in flowers and siliques revealed both shared and tissue-specific responses (**Figure 2C**). While a subset of genes was coordinately upregulated or downregulated in both organs, suggesting a conserved AGP-dependent regulatory network, most of the genes showed expression changes unique to either the flower or silique transcriptome (**Figure 2C**). Going beyond gene-to-gene comparisons, which are often insufficient for capturing shared transcriptomic responses across datasets (Olanrewaju et al., 2023), we examined the overlap in functional pathways differentially expressed in siliques and flowers. The limited number of shared pathways further emphasizes the divergent responses between these organs, likely driven by distinct developmental programs, hormonal environments, and structural requirements (Yu et al., 2020).

A gene-to-gene comparison of the directionality of gene regulation revealed a striking subset of genes that were upregulated in one tissue but downregulated in the other (**Figure 2D**). However, while a non-directional comparison of the functional pathway enrichment between the silique and flower revealed few shared pathways, when directionality was considered a clear conservation in the upregulation or downregulation of specific functional pathways emerged in both organs (**Figure 2D**), indicating that while the genes may differ, the broader regulatory strategies converge at the pathway level.

### Identification and differential expression of top genes in *galt* octuple mutant flowers

To identify key molecular processes affected by the knockout of the *GALT* genes, we focused on the top regulated genes as primary indicators of the most responsive pathways and potential mechanistic targets. These genes can serve as molecular markers to track or monitor AGP function and its downstream effects on development, cell wall integrity, and stress responses. Analysis of the most significantly differentially expressed genes in *galt* octuple mutant flowers identified both strongly downregulated and upregulated transcripts (**Figures 2B, E**; **Table 1**). Among the downregulated genes, FAD7 (AT3G11170), encoding fatty acid desaturase 7 and known to be essential to the Jasmonic acid mediated pathway for the regulation of growth and defense (Schaller et al., 2004; Wickramanayake et al., 2020), showed a log2 fold change (log2FC) of –4.4 (padj = 1.96E-261), while HMGA (AT1G14900), a chromatin-associated protein, was reduced with log2FC of –8.57 (padj = 2.65E-147). Two members of the pectin methylesterase inhibitor family (AT1G14890 and AT4G15750) were also downregulated, with log2FC values of –8.33 and –2.79, respectively. Additional suppressed genes include two galactosyltransferase family members AT4G32120 and GALT7 (AT5G53340), with log2FC values of –2.77 and –6.36. Other top downregulated genes included EXPA25 and EXPA23 (expansins), sks8, and members of the glycosyl hydrolase and alpha/beta-hydrolase superfamilies.

**Table 1:** Top significantly differentially expressed genes in *galt* octuple mutant flowers.

Status	Ensembl id	Symbol	Description	Log2FC	Padj
Top Downregulated	AT3G11170	FAD7	fatty acid desaturase 7	-4.4	1.96E-261
	AT1G14900	HMGA	high mobility group A	-8.57	2.65E-147
	AT1G14890	AT1G14890	Plant invertase/pectin methylesterase inhibitor superfamily protein	-8.33	3.73E-124
	AT4G32120	GALT8	Galactosyltransferase family protein	-2.77	7.08E-81
	AT5G47350	AT5G47350	alpha/beta-Hydrolases superfamily protein	-3.43	6.01E-70
	AT5G20630	GER3	germin 3	-1.07	6.62E-48
	AT5G17500	AT5G17500	Glycosyl hydrolase superfamily protein	-2.84	2.66E-33
	AT4G15750	AT4G15750	Plant invertase/pectin methylesterase inhibitor superfamily protein	-2.79	1.86E-23
	AT5G39300	EXPA25	expansin A25	-3.75	1.56E-21
	AT5G53340	GALT7	Galactosyltransferase family protein	-6.36	1.91E-14
	AT5G39280	EXPA23	expansin A23	-3.2	4.81E-12
	AT1G21850	sks8	SKU5 similar 8	-6.45	8.99E-12
Top Upregulated	AT5G55450	AT5G55450	Bifunctional inhibitor/lipid-transfer protein/seed storage 2S albumin superfamily protein	3.15	1.10E-55
	AT3G45860	CRK4	cysteine-rich RLK (RECEPTOR-like protein kinase) 4	6.02	1.59E-50
	AT2G18660	PNP-A	plant natriuretic peptide A	4.32	3.51E-49
	AT4G10380	NIP5;1	NOD26-like intrinsic protein 5	1.78	1.00E-43
	AT2G43570	CHI	chitinase	4.11	8.60E-39
	AT1G75040	PR5	pathogenesis-related protein 5	3.86	2.98E-37
	AT3G47480	AT3G47480	Calcium-binding EF-hand family protein	3.82	2.49E-35
	AT5G10760	AT5G10760	Eukaryotic aspartyl protease family protein	4.39	2.39E-34
	AT3G23120	RLP38	receptor like protein 38	2.91	1.83E-33
	AT2G29120	GLR2.7	glutamate receptor 2.7	3.92	2.79E-31
	AT2G32680	RLP23	receptor like protein 23	4.7	2.83E-28

In contrast, several genes exhibited strong upregulation. CRK4 (AT3G45860), encoding a cysteine-rich receptor-like kinase, showed a log2FC of 6.02 (padj = 1.59E-50). Defense and signaling-related genes such as PNP-A (AT2G18660, log2FC = 4.32), CHI (AT2G43570, log2FC = 4.11), PR5 (AT1G75040, log2FC = 3.86), and RLP23 (AT2G32680, log2FC = 4.70) were highly upregulated. Other notably upregulated genes included the lipid-transfer protein (AT5G55450), calcium-binding EF-hand protein (AT3G47480), eukaryotic aspartyl protease (AT5G10760), and glutamate receptor GLR2.7 (AT2G29120).

### Differential expression of top genes in *galt* octuple mutant siliques

The most significantly differentially expressed genes in *galt* octuple mutant siliques included both highly downregulated and upregulated transcripts (**Figures 2B, E**; **Table 2**). Among the downregulated genes, FAD7 (AT3G11170), encoding fatty acid desaturase 7, showed a log2 fold change (log2FC) of –4.93 (padj = 6.12E-178). Strong repression was also observed for AT1G14890, a pectin methylesterase inhibitor superfamily protein (log2FC = –8.24, padj = 3.85E-82), and HMGA (AT1G14900), a chromatin-associated protein (log2FC = –8.18, padj = 1.15E-41). Several galactosyltransferase family genes were significantly downregulated, including GALT8 (AT4G32120, log2FC = –3.45), GALT7 (AT5G53340, –4.24), GALT4 (AT1G27120, –2.17), and GALT3 (AT3G06440, –2.70), with highly significant adjusted p-values. Additional suppressed genes included GASA1, BG3, RGF9, and a ChaC-like family protein (AT5G26220).

**Table 2:** Top significantly differentially expressed genes in *galt* octuple mutant siliques.

Status	Ensembl id	Symbol	Description	Log2FC	Padj
Top Downregulated	AT3G11170	FAD7	fatty acid desaturase 7	-4.93	6.12E-178
	AT1G14890	AT1G14890	Plant invertase/pectin methylesterase inhibitor superfamily protein	-8.24	3.85E-82
	AT4G32120	GALT8	Galactosyltransferase family protein	-3.45	1.18E-57
	AT1G14900	HMGA	high mobility group A	-8.18	1.15E-41
	AT5G53340	GALT7	Galactosyltransferase family protein	-4.24	5.04E-17
	AT1G75750	GASA1	GAST1 protein homolog 1	-1.43	1.6E-15
	AT3G57240	BG3	beta-1,3-glucanase 3	-2.05	2.13E-11
	AT1G27120	GALT4	Galactosyltransferase family protein	-2.17	1.30E-09
	AT5G64770	RGF9	root meristem growth factor	-2.49	1.77E-09
	AT3G06440	GALT3	Galactosyltransferase family protein	-2.7	7.54E-08
	AT5G26220	AT5G26220	ChaC-like family protein	-3.34	1.80E-06
Top Upregulated	AT3G48340	CEP2	Cysteine proteinases superfamily protein	4.75	1.70E-33
	AT4G35690	AT4G35690	Hypothetical protein (DUF241)	5.91	4.07E-24
	AT1G64790	ILA	ILITYHIA	3.84	1.75E-17
	AT1G68620	AT1G68620	alpha/beta-Hydrolases superfamily protein	4.46	1.61E-15
	AT1G69490	NAP	NAC-like, activated by AP3/PI	4.65	3.81E-15
	AT2G18550	HB21	homeobox protein 21	3.82	2.94E-14
	AT4G02330	ATPMEPCRB	Plant invertase/pectin methylesterase inhibitor superfamily	4.11	5.82E-14
	AT2G36325	AT2G36325	GDSL-like Lipase/Acylhydrolase superfamily protein	5.49	9.07E-14
	AT3G22600	AT3G22600	Bifunctional inhibitor/lipid-transfer protein/seed storage 2S albumin superfamily protein	3.48	4.31E-13
	AT4G18425	AT4G18425	transmembrane protein, putative (DUF679)	2.45	8.97E-13

In contrast, multiple genes showed strong upregulation. The most highly induced gene was AT4G35690 (hypothetical protein, DUF241) with a log2FC of 5.91 (padj = 4.07E-24), followed by CEP2 (AT3G48340, log2FC = 4.75, padj = 1.70E-33). Other significantly upregulated genes included ILITHYIA (ILA) (AT1G64790, log2FC = 3.84), NAP (AT1G69490, log2FC = 4.65), HB21 (AT2G18550, log2FC = 3.82), ATPMEPCRB (AT4G02330, log2FC = 4.11), and GDSL-like lipase (AT2G36325, log2FC = 5.49). Further highly upregulated transcripts included an alpha/beta hydrolase (AT1G68620), a bifunctional inhibitor/lipid-transfer protein (AT3G22600), and a transmembrane DUF679 domain protein (AT4G18425).

### Shared gene ontology patterns between *galt* octuple mutant flowers and siliques

**Figure 3:**
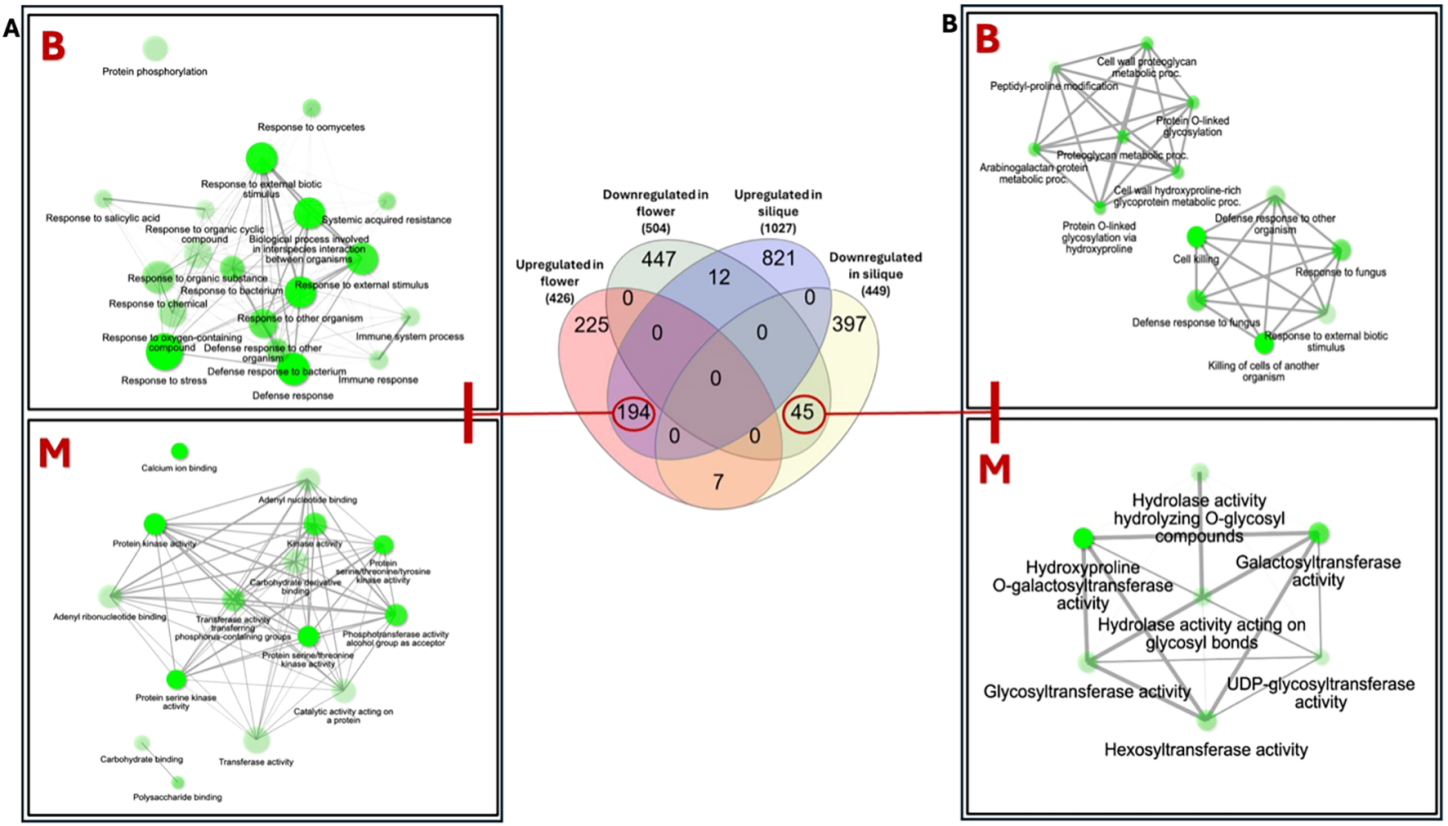
Functional enrichment analysis of shared and organ-specific differentially expressed genes in *galt* octuple mutant flowers and siliques was performed with respect to biological processes (B in red) and molecular functions (M in red). **(A)** Gene ontology (GO) enrichment analysis for the 194 genes upregulated in both flowers and siliques. **(B)** Enrichment analysis for the 45 genes downregulated in both flower and silique tissues. The size of the nodes indicates the number of genes contributing to that enrichment pathway from the dataset while the connecting lines indicate the co-interaction of the pathways. The darker the lines, the stronger the interactions. Node interaction was generated by ShinyGO v.0.86 (Ge et al., 2020).

Functional enrichment analysis of DEGs demonstrated organ-specific divergence between flowers and siliques (**Figure 3**). Among the overlapping DEGs, 19 genes displayed opposing expression patterns between the two organs (**Supplementary Figure 1**). We focused on the 194 genes commonly upregulated in both organs and the 45 genes commonly downregulated in both organs (**Figures 3A, B**) which are more plausibly the direct consequence of disrupted AGP glycosylation rather than organ-specific variability. Gene ontology enrichment analysis of the commonly upregulated genes (**Figure 3A**) showed enrichment for stress-related biological processes, including responses to bacteria, external stimuli, and stress. Enriched molecular functions included kinase activity, calcium ion binding, and phosphotransferase activity. In contrast, the 45 genes commonly downregulated in both organs were enriched for processes tied to AGP biosynthesis. Enriched biological processes included arabinogalactan-protein metabolic pathways, *O*-linked glycosylation via hydroxyproline, and the assembly of hydroxyproline-rich glycoproteins (**Figure 3B**). Corresponding molecular function terms included hydroxyproline *O*-galactosyltransferase activity, glycosyltransferase activity, and hexosyltransferase activity.

### Functional enrichment patterns specific to *galt* octuple mutant flowers

**Figure 4:**
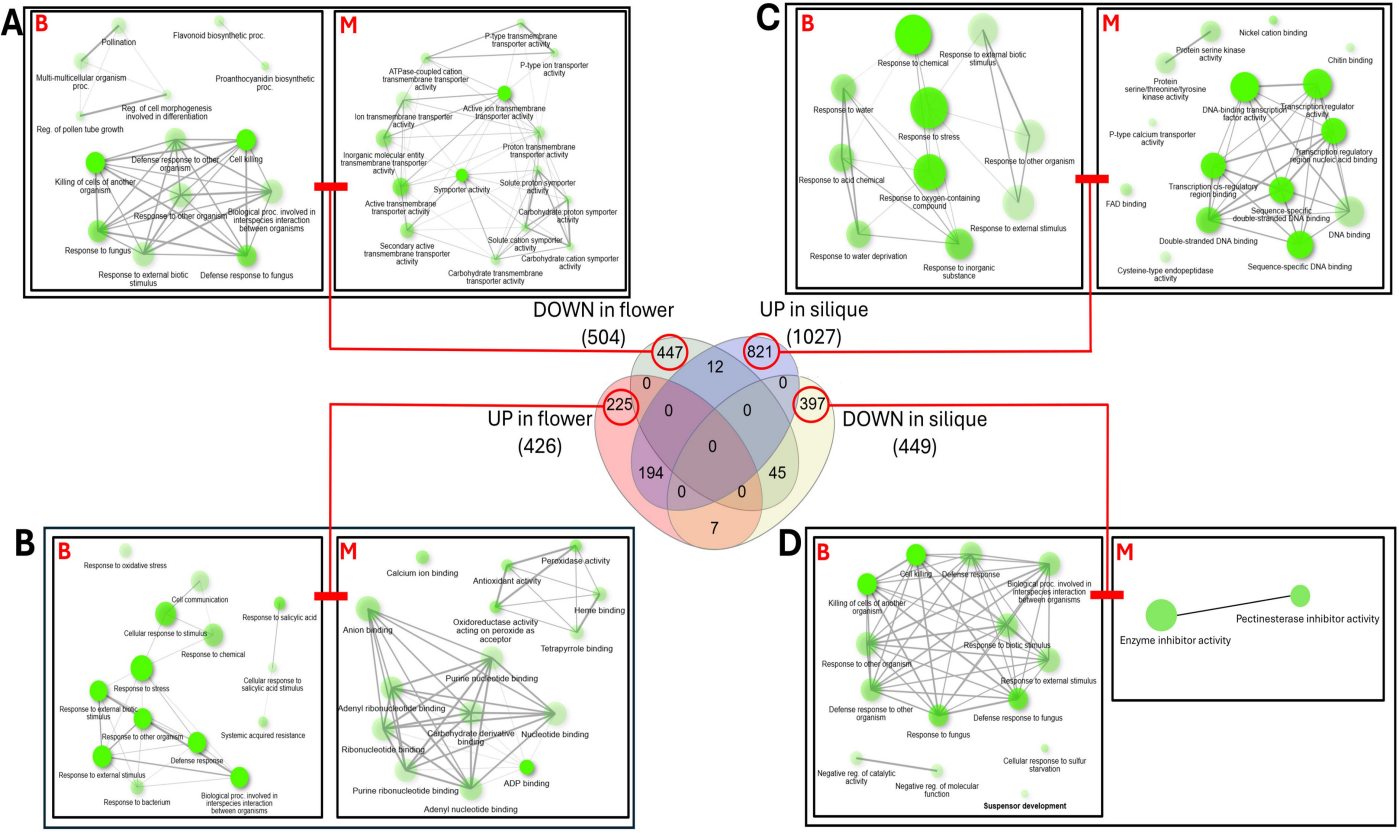
Functional enrichment analysis of differentially expressed genes specific to *galt* octuple mutant flowers and siliques was performed with respect to biological processes (B in red) and molecular functions (M in red). The central Venn diagram shows the overlap of genes differentially expressed across four categories: **(A)** Gene ontology (GO) enrichment analysis for the 447 genes downregulated in flowers only. **(B)** GO enrichment analysis for the 225 genes upregulated in flowers only. **(C)** GO enrichment analysis for the 397 genes downregulated in siliques only. **(D)** GO enrichment analysis for 827 genes upregulated in siliques only. The size of the nodes indicates the number of genes contributing to that enrichment pathway from the dataset while the connecting lines indicate the co-interaction of the pathways. The darker the lines, the stronger the interactions. Node interaction was generated by ShinyGO v.0.86 (Ge et al., 2020).

The pathways that are enriched by the differentially expressed genes specifically in flowers are involved in various biological processes, molecular functions and cellular components. The upregulated genes in mutant flowers (**Figure 4B**) indicate increased metabolic activity and an oxidative stress response, with enhanced expression of peroxidases, antioxidant enzymes, and calcium-binding proteins, which play crucial roles in cell signaling and stress adaptation (Gadjev et al., 2006). High expression of cell wall-modifying enzymes, such as cellulase and glucan endo-1,3-beta-D-glucosidase, suggests compensatory mechanisms affecting pollen tube growth and flower development. Conversely, downregulated genes (**Figure 4A**) are involved in transmembrane transport, carbohydrate metabolism, and flavonoid biosynthesis which disrupts nutrient uptake, energy availability, and floral pigmentation, potentially disrupting pollination success and reproduction. The reduced activity of P-type ATPases and carbohydrate transporters may further weakens ion homeostasis and metabolic balance and could lead to delay in flowering time as well as an alteration in flower morphology (Andrés et al., 2020; Haruta et al., 2015; Mulet et al., 2020).

### Functional enrichment patterns specific to *galt* octuple mutant siliques

The 397 genes downregulated specifically in siliques (**Figure 4D**) were enriched for biological processes like flavonoid biosynthesis and response to sulfur starvation. The downregulation of genes involved in flavonoid biosynthesis observed specifically in mutant siliques can lead to reduced flavonoid production, affecting pigmentation, UV protection, and pathogen defense, making plants more susceptible to environmental stress. Similarly, downregulation of sulfur starvation response genes may impair sulfur uptake and assimilation, leading to growth defects such as chlorosis and reduced seed yield. The reduced seed set observed in the *galt* octuple mutant (Kaur et al., 2023) may be attributed to the disruption of early embryo development caused by the loss of suspensor-related gene expression. This is due to the fact that the suspensor, which is the embryonic region, connects the embryo to the seed coat (Kawashima and Goldberg, 2010). Reduced pectin methylesterase inhibitor (PMEI) expression weakens cell wall integrity, increasing susceptibility to mechanical stress and pathogens (Okawa et al., 2023). Conversely, 821 specifically upregulated genes in siliques (**Figure 4C**) are involved in protein-ligand interactions, calcium transport, and transcriptional regulation which suggests a stress-induced compensatory response to maintain cellular function in mutant siliques (Gadjev et al., 2006).

### Gene enrichment clustering analysis of DEGs in *galt* octuple mutant flowers and siliques

**Figure 5:**
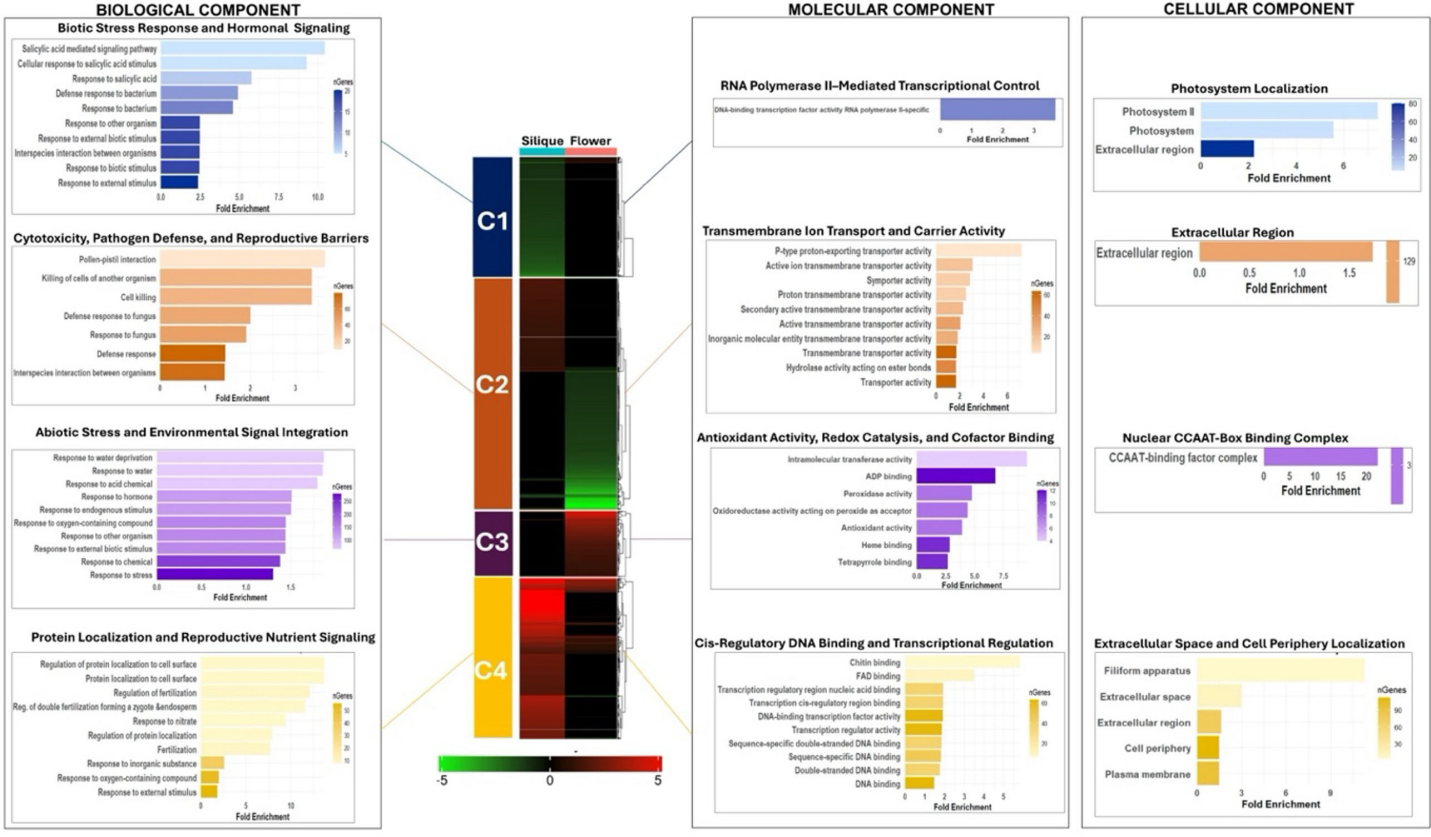
Functional-clustering heat map of the gene enrichment analysis. Differentially expressed genes from galt octuple mutants were first hierarchically clustered based on shared gene ontology (GO) enrichment (padj < 0.05), yielding four functional modules (C1-C4, center heat map; green = downregulated, red = upregulated). For each module, bar charts (left = biological process component; center = molecular function component; right = cellular component) display the top GO terms (x-axis = fold-enrichment; color scale = gene count).

To gain a systems-level understanding of how AGP glycosylation deficiency modulates plant transcriptomes, we conducted a gene set enrichment-based clustering analysis, grouping differentially expressed genes not by expression magnitude or direction alone, but by the functional similarity of their enriched biological, molecular, and cellular roles (Ge et al., 2018). This approach enabled the identification of co-regulated gene groups that operate within shared biological frameworks, even across organs with divergent transcriptomic landscapes. The resulting functional clusters revealed four major modules of interest, each representing a distinct physiological axis perturbed by abnormal AGP glycosylation (**Figure 5**).

Cluster 1 consisted primarily of genes downregulated in siliques, enriched for biological processes including salicylic acid–mediated signaling, bacterial defense response, and inter-organismal communication, as well as molecular functions related to RNA polymerase II–associated transcriptional regulation and cellular components such as the photosystem and extracellular region (**Figure 5, C1**). Cluster 2 contained genes upregulated in siliques and downregulated in flowers, with enrichment for pollen–pistil interaction, pathogen defense, cytotoxicity, proton-exporting ATPase activity, symporter activity, and extracellular localization (**Figure 5, C2**). Cluster 3 comprised genes upregulated in flowers and downregulated in siliques, enriched for biological processes associated with water deprivation, oxidative response, and hormonal stimulus, together with molecular functions including peroxidase activity and localization to the nuclear CCAAT-binding complex (**Figure 5, C3**). Cluster 4 included genes upregulated in both organs, enriched for protein localization to the cell surface, double-fertilization processes, nitrate-responsive signaling, cis-regulatory DNA-binding activity, and cellular structures associated with the filiform apparatus and other extracellular interfaces (**Figure 5, C4**).

### Gene set enrichment analysis (GSEA) in *galt* octuple mutant flowers and siliques

**Figure 6:**
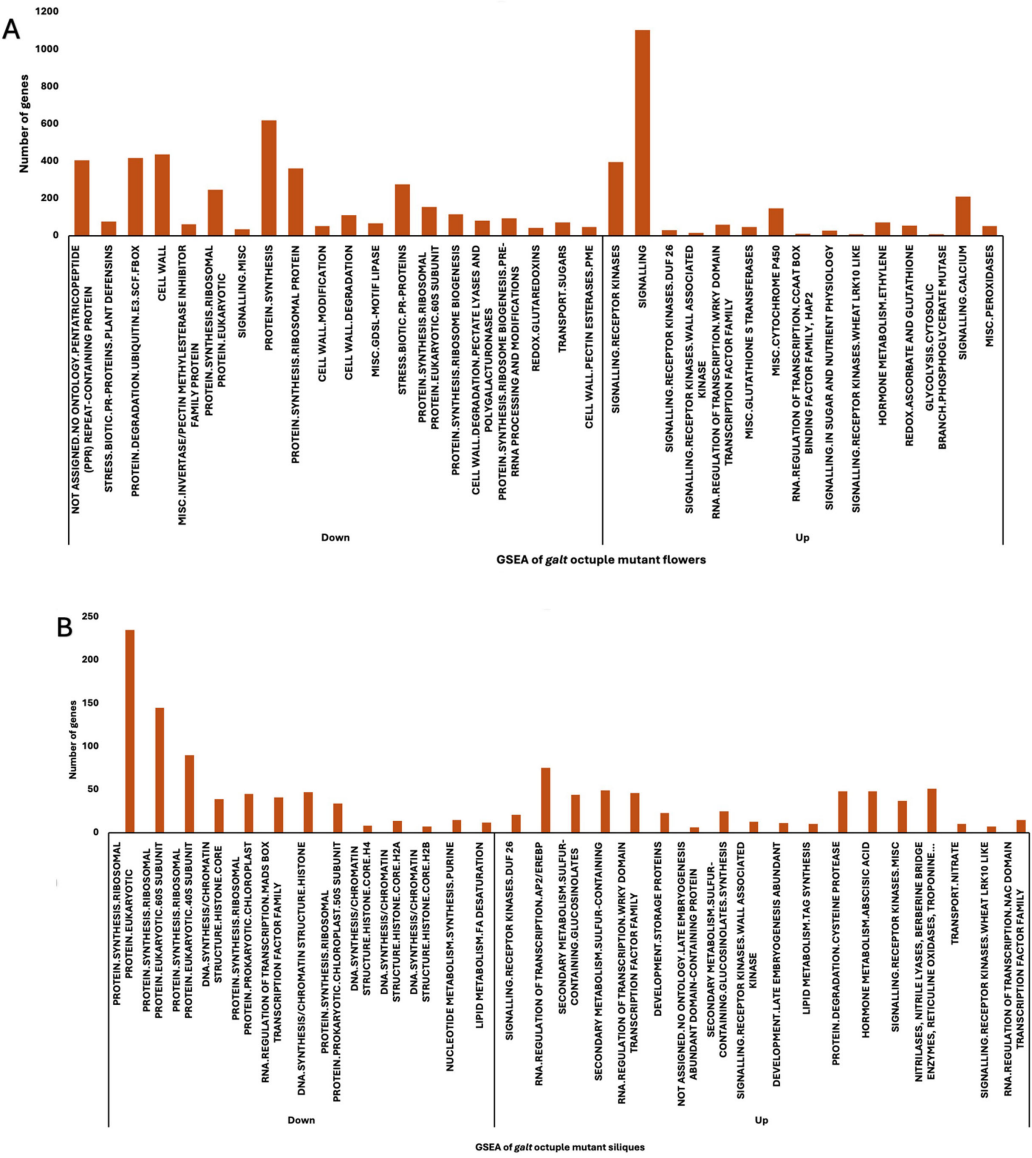
Gene set enrichment analysis (GSEA) showing enriched pathways and the number of genes involved in each gene set for flowers **(A)** and siliques **(B)**. Genes related to RNA regulation, receptor kinases, cell wall modification, and stress response show significant transcriptional changes in galt octuple mutant flowers and siliques.

To gain a broader, pathway-level understanding of these changes, we employed the gene set enrichment analysis to study the expression patterns of significantly differentially expressed genes in *galt* octuple mutant flowers and siliques using MapMan functional categorization (**Figure 6**). This approach allowed for the identification of entire predefined biological pathways that were systematically enriched for upregulation or downregulation across the full spectrum of gene expression, rather than focusing solely on genes exceeding a significance threshold. In the octuple mutant flowers, enriched downregulated categories included pentatricopeptide repeat-containing proteins, protein degradation (ubiquitin-related), PMEI proteins, cell wall organization, ribosomal proteins, and DNA synthesis/chromatin structure. Additional categories such as cell wall-modifying enzymes, biotic stress-related proteins, cell wall degradation, pectate lyases and polygalacturonase and genes linked to regulation of cell wall pectinesterases (**Figure 6A**) were also represented among the downregulated gene sets. Upregulated categories in the mutant flowers included many genes associated with signaling receptor kinases, particularly DUF26 domain kinases, WAK-like kinases, and LRK10-like kinases. Genes involved in glutathione transferases, transcription regulation, and hormone-responsive elements and peroxidases (**Supplementary Table 1**), were also enriched among the upregulated sets.

In siliques (**Figure 6B**), downregulated gene sets were dominated by cytosolic and ribosomal proteins, especially eukaryotic 40S and 60S subunits, along with genes related to histone core components, DNA synthesis, and RNA-binding proteins. Several stress-related and chromatin-associated gene sets also appeared in the downregulated list. Upregulated gene sets in siliques included those associated with secondary metabolism, embryogenesis, storage protein synthesis (**Supplementary Table 2**), lipid metabolism, and receptor kinase signaling (**Table 3**) pathways. Like flowers, siliques showed enrichment of DUF26 domain kinases, transcription factors (**Supplementary Table 3**), and transport-related proteins. These profiles indicate distinct patterns of gene category enrichment across the two reproductive organs.

**Table 3:** The upregulation of receptor kinase and wall associated kinase gene sets in flowers and siliques.

The upregulation of receptor kinase and wall associated kinase gene sets in flowers							
Receptor kinases				Wall associated kinases			
Gene_ID	Symbol	log2FC	padj	Gene_ID	Symbol	log2FC	padj
AT4G28670	AT4G28670	-0.895	0.29	AT2G23450	WAKL14	0.15	0.71
AT2G33330	PDLP3	-0.162	0.2	AT3G53840	AT3G53840	0.17	0.84
AT2G01660	PDLP6	-0.04	0.97	AT4G31100	AT4G31100	0.22	0.77
AT2G01660	PDLP6	-0.025	0.98	AT1G16130	WAKL2	0.89	0.1
AT4G11521	AT4G11521	0.51	0.58	AT1G16110	WAKL6	1.01	1.46E-05
AT4G23300	CRK22	0.7	0.11	AT5G66790	AT5G66790	1.45	0.049
AT4G23250	EMB1290	0.9	0.07	AT1G69730	AT1G69730	1.55	2.04E-08
AT1G70530	CRK3	0.9	0.24	AT1G79680	WAKL10	1.79	0.03
AT4G11530	CRK34	1.2	0.0013	AT1G21270	WAK2	2.62	7.69E-19
AT4G23220	CRK14	2.49	8.05E-14	AT1G21250	WAK1	2.77	2.79E-19
AT4G23260	CRK18	2.52	1.17E-06				
AT4G11890	ARCK1	2.68	3.96E-08				
AT4G23260	CRK18	3.2	4.82E-09				
The upregulation of receptor kinase and wall associated kinase gene sets in siliques							
Signalling. Receptor kinases.DUF26				Wall associated receptor kinases			
Gene_ID	Symbol	log2FC	padj	Gene_ID	Symbol	log2FC	padj
AT4G23300	CRK22	-0.34	0.48	AT1G79670	WAKL22	0.37	0.45
AT2G33330	PDLP3	-0.31	0.26	AT1G21270	WAK2	0.46	0.43
AT2G01660	PDLP6	0.33	0.39	AT1G69730	AT1G69730	1.17	0.03
AT4G23180	CRK10/CRK7	1.67	0.063	AT2G23450	WAKL14	1.43	0.001
AT4G11530	CRK34	1.73	0.022	AT5G66790	AT5G66790	1.88	0.008
AT4G23250	EMB1290	2.18	0.00023	AT1G16110	WAKL6	1.93	0.00032
AT4G23260	CRK18	2.59	0.0024	AT1G21250	WAK1	2.31	0.00074
AT4G11521	AT4G11521	2.75	0.02	AT1G16130	WAKL2	3.53	0.014
AT4G23220	CRK14	3.45	1.68E-06	AT1G21240	WAK3	4.45	3.77E-05
AT4G11890	ARCK1	3.97	0.00061	AT1G79680	WAKL10	6.16	0.00039
AT4G23230	CRK15	4.13	8.55E-05				
AT4G23150	CRK2	4.22	0.001				

### Expression of receptor kinase and wall-associated kinase gene sets

Gene set enrichment analysis (**Figures 6A, B**) showed the upregulation of receptor kinases and wall-associated kinases gene sets in the galt octuple mutant flowers and siliques (**Table 3**). However, within these gene sets, expression analysis (**Table 3**) revealed both upregulation and downregulation of specific genes belonging to receptor kinase and wall-associated kinase (WAK) families in the galt octuple mutant. In flowers, among the receptor kinases, CRK18 (AT4G23260) showed the highest upregulation with a log2FC of 3.20 and a padj of 4.82E-09, followed by ARCK1 (AT4G11890) at log2FC 2.68 (padj = 3.96E-08), and CRK14 (AT4G23220) at log2FC 2.49 (padj = 8.05E-14). Other moderately upregulated receptor kinases include CRK34 (log2FC = 1.20, padj = 0.0013) and EMB1290 (log2FC = 0.90, padj = 0.07). In contrast, a few receptor kinases were downregulated, including AT1G19090 (log2FC = -1.083, padj = 0.09) and AT4G28670 (log2FC = -0.895, padj = 0.29).

For the WAK gene set, several members were significantly upregulated. WAK1 (AT1G21250) and WAK2 (AT1G21270) displayed strong upregulation with log2FC values of 2.77 and 2.62, and padj values of 2.79E-19 and 7.69E-19, respectively. Other upregulated genes include WAKL10 (log2FC = 1.79, padj = 0.03), WAKL6 (log2FC = 1.01, padj = 1.46E-05), and AT5G66790 (log2FC = 1.45, padj = 0.049). A few WAK-related genes such as AT2G23450 (log2FC = -0.37, padj = 0.67) showed mild downregulation or no significant change. In Siliques, among the CRKs, CRK14 (AT4G23220) showed a strong upregulation (log2FC = 3.45, padj = 1.68E-06), alongside CRK15, CRK2, ARCK1, and CRK18, all exhibiting greater than 2-fold expression increases. Notably, EMB1290 (AT4G23250) was also upregulated (log2FC = 2.18, padj = 0.00023), suggesting a broader activation of DUF26-RLK signaling.

Similarly, genes encoding wall-associated kinases were markedly upregulated, with WAK3 (AT1G21240) and WAKL10 (AT1G79680) showing log2FC values of 4.45 (padj = 3.77E-05) and 6.16 (padj = 0.00039), respectively. Other significantly induced WAK family genes included WAK1, WAK2, WAKL2, and WAKL6, all with log2FC > 1.9 and padj < 0.01. These genes are known to mediate wall integrity sensing and signal transduction in response to biotic and mechanical triggers. The coordinated induction of CRKs and WAKs supports a model in which loss of AGP glycosylation activates cell surface surveillance systems, likely as a compensatory mechanism to maintain wall structure and stress responses during reproductive development.

### Differential expression of cell wall organization genes in *galt* octuple mutant flowers

GSEA in flowers showed a significant downregulation of a cell wall organization gene set (**Figure 6A**), however, a mixed pattern of regulation was observed among the genes associated with cell wall organization of *galt* octuple mutant flowers, as revealed in **Table 4**. The most strongly downregulated genes included *AT5G44840* (log_2_;FC = –7.02, padj = 1.58e-08), *EXPB4* (*AT2G45110*, –5.97, 2.01e-06), and *AT4G33840* (–4.48, 5.63e-09). Other members of the expansin family such as *EXPA25* (*AT5G39300*, –3.76, 8.41e-22), *EXPA23* (*AT5G39280*, –3.21, 2.85e-12), and *EXPA16* (*AT3G55500*, –1.57, 0.000195) also showed reduced expression. The most strongly upregulated gene was *PNP-A* (*AT2G18660*, log_2_FC = 4.313, padj = 2.54e-48), followed by *FUT4* (*AT2G15390*, 2.514, 0.019), *COBL5* (*AT5G60950*, 2.47, 1.77e-12), and *AGP5* (*AT1G35230*, 2.329, 1.34e-13). Additional upregulated genes included *EXT3*, *XTR6*, *PMEPCRF*, *PGAZAT*, *EXLB1*, *COBL6*, *XTH19*, *AGP40*, and *LRX2*, with fold changes between 1.06 and 2.23.

**Table 4:** Genes involved with downregulated cell wall pathways in *galt* octuple mutant flowers.

Cell wall				
Gene_ID	Symbol	log2FC	padj	Description
AT5G44840	AT5G44840	-7.02	1.58E-08	Pectin lyase-like superfamily protein
AT2G45110	EXPB4	-5.97	2.01E-06	expansin B4
AT4G33840	AT4G33840	-4.48	5.63E-09	Glycosyl hydrolase family 10 protein
AT5G39300	EXPA25	-3.76	8.41E-22	expansin A25
AT5G39280	EXPA23	-3.21	2.85E-12	expansin A23
AT4G03930	AT4G03930	-1.98	7.51E-07	Plant invertase/pectin methylesterase inhibitor superfamily
AT3G55500	EXPA16	-1.57	0.000195	expansin A16
AT4G35670	AT4G35670	-1.56	1.81E-08	Pectin lyase-like superfamily protein
AT5G25550	AT5G25550	-1.54	1.50E-24	Leucine-rich repeat (LRR) family protein
AT3G48950	AT3G48950	-1.3	0.006	Pectin lyase-like superfamily protein
AT3G14040	AT3G14040	-1.3	8.66E-09	Pectin lyase-like superfamily protein
AT1G56620	AT1G56620	-1.25	0.024	Plant invertase/pectin methylesterase inhibitor superfamily protein
AT3G07850	AT3G07850	-1.24	3.44E-08	Pectin lyase-like superfamily protein
AT5G07410	AT5G07410	-1.2	1.85E-07	Pectin lyase-like superfamily protein
AT4G07960	CSLC12	-1.2	0.0047	Cellulose-synthase-like C12
AT3G07620	AT3G07620	-1.13	3.13E-05	glycosyltransferase
AT4G31370	FLA5	-1.03	4.50E-07	FASCICLIN-like arabinogalactan protein 5
AT3G20865	AGP40	1.068	0.00042	arabinogalactan protein 40
AT4G30290	XTH19	1.116	0.05	xyloglucan endotransglucosylase/hydrolase 19
AT1G09790	COBL6	1.163	0.011	COBRA-like protein 6
AT3G27400	AT3G27400	1.289	0.013	Pectin lyase-like superfamily protein
AT4G17030	EXLB1	1.293	0.0002	expansin-like B1
AT2G41850	PGAZAT	1.384	0.034	polygalacturonase ADPG2-like protein
AT5G53370	PMEPCRF	1.477	4.13E-09	pectin methylesterase PCR fragment F
AT1G21310	EXT3	1.486	0.0025	extensin 3
AT4G25810	XTR6	1.505	0.0063	xyloglucan endotransglycosylase 6
AT1G62440	LRX2	2.228	0.012	leucine-rich repeat/extensin 2
AT1G35230	AGP5	2.329	1.34E-13	arabinogalactan protein 5
AT5G60950	COBL5	2.47	1.77E-12	COBRA-like protein 5
AT2G15390	FUT4	2.514	0.019	fucosyltransferase 4
AT2G18660	PNP-A	4.313	2.54E-48	plant natriuretic peptide A

### Altered expression of genes involved in AGP glycosylation

Functional analysis of differentially expressed genes in *galt* octuple mutant flowers and siliques, using integrated annotations from the carbohydrate-active enzymes (CAZy) database and Plant Gene Set annotation database (PlantGSAD), revealed a marked disruption in the expression of genes encoding enzymes responsible for AGP glycosylation. This highlights the central role of glycosyltransferases and other carbohydrate-active enzymes in maintaining AGP structure and function. Specifically, 26 glycosyltransferase-related genes were significantly downregulated in flowers and siliques (**Table 5**), including members of the GT31 family, which are known to catalyze the addition of galactose residues to hydroxyproline-rich protein backbones during AGP biosynthesis (Basu et al., 2015; Ogawa-Ohnishi and Matsubayashi, 2015). These changes extend beyond the *GALT* family to include both well-characterized and uncharacterized glycosyltransferases expressed during reproductive development. Notably, the expression of *CAGE1* and *CAGE2*, two Golgi-localized glycosyltransferases implicated in both β-1,3-galactan synthesis and cellulose biosynthesis was markedly reduced, suggesting a broader impact on cell wall carbohydrate assembly (Nibbering et al., 2020, 2022). This is consistent with a study that have shown defects in galactosyltransferases resulted in reduced cellulose biosynthesis, which in causes cell wall defects (Nibbering et al., 2022)

**Table 5:** Differential regulation of AGP-related glycosyltransferases in *galt* octuple mutant flowers and siliques.

Glycosyltransferase family (Flowers)									
Gene_ID	Symbol	log2FC	padj	Gene_ID	Symbol	log2FC	padj
AT5G53340	GALT7	-8.66	4.92e-14	AT1G01570	CAGE1	0.002	0.998
AT4G21060	GALT2	-2.04	0.04	AT3G14960	AT3G14960	0.05	0.849
AT3G06440	GALT3	-1.87	1.24e-09	AT3G11420	AT3G11420	0.06	0.812
AT1G27120	GALT4	-1.61	5.35e-14	AT1G74800	GALT5	0.06	0.738
AT1G07850	AT1G07850	-1.29	5.06e-14	AT4G15240	AT4G15240	0.09	0.785
AT1G53290	GALT9	-0.12	0.52	AT5G41460	AT5G41460	0.10	0.806
AT1G33250	AT1G33250	-0.07	0.79	AT2G26100	AT2G26100	0.16	0.849
AT2G32430	AT2G32430	-0.06	0.895	AT4G00300	AT4G00300	0.20	0.260
AT5G57500	AT5G57500	-0.06	0.94	AT1G11730	AT1G11730	0.22	4.03E-01
AT1G32930	GALT31A	-0.03	0.922	AT1G77810	AT1G77810	0.43	0.040
AT4G23490	AT4G23490	-0.02	0.961	AT4G26940	CAGE2	0.45	0.00044
Glycosyltransferase family (Siliques)									
AT5G53340	GALT7	-7.63	3.74E-10	AT1G74800	GALT5	0.03	0.89
AT3G06440	GALT3	-2.71	1.02E-07	AT4G23490	AT4G23490	0.06	0.82
AT1G27120	GALT4	-2.17	8.15E-10	AT4G15240	AT4G15240	0.07	0.87
AT4G32110	AT4G32110	-1.14	0.006	AT1G01570	CAGE1	0.14	0.92
AT5G41460	AT5G41460	-0.43	0.12	AT4G26940	CAGE2	0.30	0.29
AT1G32930	GALT31A	-0.37	0.03	AT1G33250	AT1G33250	0.33	0.08
AT5G57500	AT5G57500	-0.24	0.80	AT1G53290	GALT9	0.41	0.03
AT1G11730	AT1G11730	-0.16	0.83	AT1G77810	AT1G77810	0.85	0.18
AT4G00300	AT4G00300	-0.08	0.71	AT3G11420	AT3G11420	0.95	0.01
AT3G14960	AT3G14960	-0.05	0.88	AT4G21060	GALT2	1.70	0.03
AT2G32430	AT2G32430	-0.01	0.98				

In addition, 11 genes belonging to the glycoside hydrolase (GH) family were upregulated (**Supplementary Table 4**) in both organs. While certain glycoside hydrolases (GH43 family) localized to the apoplast or Golgi have been shown to play a quality control role in AGP biosynthesis by removing aberrant β-1,3-galactan chains from growing glycans (Leszczuk et al., 2023; Nibbering et al., 2020; Tryfona et al., 2012), the GH19 family which was observed to be upregulated here are not directly linked to AGP synthesis and contains enzymes that are biochemically characterized as chitinases and lysozymes (Zhou et al., 2024). Many AGPs have been suspected to be substrates for chitinases (Showalter, 2001; van Hengel et al., 2001). These GH19 family enzymes are known to be associated with plant pathogenesis (Orlando et al., 2021).

## Discussion

### Impacts of transcriptional reprogramming on development and reproduction

Despite the knockout of eight primary galactosyltransferase genes (*GALT2–GALT9*), the octuple mutant remains viable, likely due to gene/enzymatic redundancy within the GT31 family or other uncharacterized glycosyltransferases, combined with extensive transcriptional reprogramming as identified in our RNA-seq data. The top significantly upregulated and downregulated genes in flowers and siliques of *galt* octuple mutants are summarized in **Tables 1**, **2**, respectively. Common themes emerge across both tissues, highlighting the roles of AGPs in cell wall integrity, lipid signaling, chromatin remodeling, and defense responses. Downregulated genes in both tissues include *FAD7* (*AT3G11170*), encoding fatty acid desaturase 7 crucial for trienoic fatty acid biosynthesis, membrane fluidity, and stress responses (McConn and Browse, 1996; Wickramanayake et al., 2020). This suggests that AGPs modulate lipid-derived signaling at the cell wall-plasma membrane interface, impacting pollen viability, silique expansion, and hormone pathways (He and Ding, 2020; Hoffmann et al., 2020; Qian et al., 2025). *HMGA* (*AT1G14900*), encoding a chromatin-associated protein (Reeves, 2001), is also repressed, implying reduced chromatin remodeling that hinders growth-associated gene expression (Vignali and Marracci, 2020). The downregulation of pectin methylesterase inhibitor genes (PMEIs, e.g., *AT1G14890*, *AT4G15750*, *AT4G02330*) may indicate disrupted pectin remodeling, leading to weakened cell wall integrity (Chebli and Geitmann, 2018; Wolf et al., 2009), expansion, and pathogen defense (Lionetti et al., 2007; Peaucelle et al., 2012; Pei et al., 2024; Wolf et al., 2009; Wormit and Usadel, 2018). Transcriptomic analyses reveal a marked downregulation of gene set critical for pollen tube elongation and successful fertilization. The reduced expression of these genes might have led to increased pectin demethylesterification and compromised wall structure during flower and silique development, supporting the hypothesis that AGPs interact with or co-regulate pectin-modifying enzymes, contributing to cell wall dynamics. Expansin genes (*EXPA23*, *EXPA25*) are downregulated in flowers, impairing cell wall loosening and growth processes, consistent with the established role of AGPs in cell expansion and development (Cosgrove, 2018). Multiple galactosyltransferase genes e.g., *GALT7* (*AT5G53340*), *GALT8* (*AT4G32120*), *GALT3* (*AT3G06440*), *GALT4* (*AT1G27120*) are repressed, confirming disrupted AGP glycosylation due to the mutation of the *GALT* genes (Zhang, 2009). Similarly, *sks8* (*AT1G21850*), encoding a SKU5-similar protein with AGP-like features (Sedbrook et al., 2002), is strongly downregulated, highlighting the interconnectedness of glycosylated proteins in cell wall integrity and signaling. Additionally, genes encoding a beta-1,3-glucanase and a member of the glycoside hydroxylase family implicated in defense and remodeling of the callose matrix, and *AT5G26220* are also downregulated, supporting the view that AGPs modulate not only wall structure but also influence developmental signaling peptides and enzymes involved in dynamic cell wall restructuring.

In flowers, the high upregulation of *CRK4* (*AT3G45860*), *RLP23*, and *RLP38*, encoding receptor-like kinases and proteins involved in pathogen perception, indicates enhanced surveillance and stress signaling. This may reflect attempts to reestablish floral developmental balance via stress-related transcriptional reprogramming (Cui et al., 2022). The upregulation of the expression of calcium-dependent protein kinases (CDPKs) and calmodulin-binding proteins, key components of calcium-mediated signaling cascades, implicates a broader regulatory role for AGPs in coordinating pollen tube guidance and fertilization. These findings align with previous reports that highlight calcium as a central second messenger in plant reproduction, where spatial and temporal oscillations in calcium levels, decoded by CDPKs and calmodulin complexes, are essential for proper pollen tube navigation and sperm delivery (Steinhorst and Kudla, 2013; Yang et al., 2021). *RLP23* is known to mediate immunity by recognizing pathogen-derived peptides (Albert et al., 2015), suggesting that AGPs may normally suppress or buffer immune responses under non-stressed conditions. Other upregulated genes, such as *CHI* (*AT2G43570*) and *PR5* (*AT1G75040*), encode pathogenesis-related proteins involved in fungal resistance and defense priming. Their increased expression indicates that AGPs may modulate immune homeostasis, and that *galt* mutants exhibit a heightened defense response with the reduced glycosylation in AGPs. The upregulation of *PNP-A* (*AT2G18660*), encoding a signaling peptide with natriuretic activity, reflects broader physiological stress responses, potentially mimicking pathogen attack or osmotic imbalance (Turek et al., 2014). The upregulation of *AT3G47480*, encoding a calcium-binding EF-hand protein, further links the AGP network to calcium signaling and cell wall integrity sensing (Lamport and Várnai, 2013). Additionally, *AT2G29120*, *AT5G10760*, and *AT5G55450* are also upregulated, suggesting a complex interplay of signaling pathways in response to AGP deficiency. On the other hand, the upregulation of the boric acid transporter gene NIP5;1 may reflect an adaptive response to preserve cell wall integrity under conditions of impaired glycosylation. Recent studies emphasize that boron’s most clearly established structural role is in cross-linking the pectic polysaccharide rhamnogalacturonan-II (RG-II) via borate diester bridges, which are critical for maintaining cell wall architecture and proper plant development (Chu et al., 2025; Matthes et al., 2020). *In vitro* studies demonstrate that RG-II dimerization via boron is otherwise inefficient without chaperone activity, a role recently attributed to specific AGPs such as AGP31 and its histidine-rich peptides (Sanhueza et al., 2022). NIP5;1 is localized to the plasma membrane and its expression is rapidly upregulated in response to boron deficiency, functioning to facilitate boric acid influx into developing tissues (Matthes et al., 2020). Therefore, enhanced NIP5;1 expression in reproductive structures may represent a feedback mechanism aiming to rescue boron-mediated processes such as RG-II cross-linking, whose disruption compromises cell expansion, cell division, and tissue morphogenesis.

In siliques, the upregulation of *AT4G35690*, encoding a DUF241 domain-containing protein, hints at the activation of poorly characterized or novel stress-responsive mechanisms. Likewise, *GDSL-like lipase* gene (*AT2G36325*), involved with the catalysis of acyltransfer or hydrolase reactions with lipid and non-lipid substrates, and the *alpha/beta-hydrolase* gene (*AT1G68620*), associated with lipid and cuticle integrity, are upregulated. *CEP2*, *ILA* (*AT1G64790*), and *GASA1*, which are associated with proteolysis, immune signaling, and cell elongation, respectively, potentially represent compensatory pathways to mitigate the loss of AGP function (Bauer et al., 2021; Hierl et al., 2014; Zhang et al., 2022). *NAP* (*AT1G69490*), encoding a NAC transcription factor involved in senescence and downstream of floral organ identity genes (Guo and Gan, 2006), is also upregulated, suggesting that AGP dysfunction might accelerate developmental transitions or trigger premature tissue maturation. The induction of *HB21* (*AT2G18550*), a homeobox gene involved in growth repression, further supports this hypothesis. *ATPMEPCRB* (*AT4G02330*), another PMEI-like gene, is also upregulated, which is notable given the downregulation of similar family members in the same tissue, suggesting a potential divergence in PMEI function or spatial regulation of pectin-related enzymes in siliques. Finally, the upregulation of *AT4G18425*, encoding a protein with a DUF1680 domain, and AT3G22600, encoding the bifunctional inhibitor/lipid-transfer protein (*AT3G22600*), suggest complex changes in hormone metabolism, signaling, and lipid transport, potentially reflecting altered cell-cell communication and resource allocation in developing siliques. Altogether, these findings reinforce the idea that AGPs, via their glycosylation and localization in the extracellular matrix mediate crosstalk between development, metabolism, and defense. In siliques, where tissue differentiation and seed maturation occur, the disruption of AGP glycosylation leads to profound transcriptional changes, particularly in genes associated with cell wall metabolism, lipid signaling, and developmental regulation.

### Cluster-based analysis reveals organ-specific compensatory and defense strategies

Cluster 1 comprised genes predominantly downregulated in siliques and enriched for salicylic acid-mediated signaling, bacterial defense response, and inter-organismal communication at the biological level, alongside RNA polymerase II-specific transcriptional regulators and proteins localized to the photosystem and extracellular space. The simultaneous repression of immune signaling, transcriptional activation, and chloroplast-associated localization suggests a broad suppression of basal defense readiness and stress-responsive gene networks in siliques of the galt octuple mutant. Given the reported role of AGPs in cell wall-associated immune perception and hormone modulation, their altered glycosylation may attenuate wall-derived signaling cues, leading to a muted salicylic acid defense program and reduced chloroplast-to-nucleus communication (Basu et al., 2016; Přerovská et al., 2022). This coordinated downregulation reflects a silique-specific vulnerability where the loss of AGP function compromises both extracellular defense and plastid-associated stress response systems.

Cluster 2 contained genes predominantly upregulated in silique and downregulated in flower tissue and enriched for biological processes related to pollen-pistil interaction, pathogen defense, and cytotoxicity, alongside molecular functions involved in ion transport, including proton-exporting and symporter activities, and localization to the extracellular region. These signatures point to a silique-specific activation of ion-flux–driven signaling at the cell surface, a mechanism that can simultaneously facilitate nutrient mobilization for developing seeds and strengthen outward-facing defense barriers (Zuo et al., 2025). The concurrent repression of the same repertoire in flowers suggests that, in the absence of properly glycosylated AGPs, floral tissues dampen costly cytotoxic and transporter programs, possibly to avoid self-incompatibility or oxidative damage, whereas siliques compensate by escalating extracellular surveillance and transport capacity. Such organ-opposite regulation is consistent with reports that AGP perturbation can differentially modulate receptor-like kinase signaling, ion gradients, and cell-cell recognition during reproduction (Foubert-Mendes et al., 2025; Pereira et al., 2016), indicating the context-dependent strategies plants deploy to maintain reproductive success and pathogen resistance when cell wall glycoprotein integrity is compromised.

Cluster 3 gene sets were activated in flowers and repressed in siliques, and their enrichment profile centers on abiotic-stress perception (water deprivation, oxidative and hormonal cues), antioxidant and peroxidase activities, and localization to the nuclear CCAAT-binding factor complex. The flower-specific upregulation of this redox-detoxification module suggests that floral tissue mounts a nucleus-driven oxidative-stress program to compensate for AGP-linked cell wall defects, whereas siliques suppress the same circuitry, perhaps relying instead on alternative, nutrient-focused defenses noted in other clusters. Such organ-opposite deployment of ROS-buffering genes echoes observations that AGP perturbations elicit context-dependent redox signaling, with floral organs often exhibiting heightened oxidative control to safeguard reproductive viability (Padhiar et al., 2025; Zinta et al., 2016).

Cluster 4 contains functional gene sets upregulated in both siliques and flowers, with top enrichment for protein localization to the cell surface, double-fertilization, and nitrate-responsive signaling, alongside cis-regulatory DNA-binding activities and products targeted to the filiform apparatus and other extracellular interfaces. This profile indicates a concerted effort to bolster reproductive nutrient exchange and cell-to-cell communication in response to altered AGP glycosylation, likely ensuring that gamete recognition and nutrient allocation proceed despite impaired cell wall glycoprotein architecture, a role consistent with the involvement of AGPs in pollen-tube guidance and extracellular signal presentation (Foubert-Mendes et al., 2025; Pereira et al., 2016).

Reduction of glycosylation by the disruption of galactosylation induces transcriptional reprogramming that alters major pathways critical for cellular function.

In flowers, the downregulated gene set notably includes 406 genes related to pentatricopeptide repeat-containing proteins, which are critical for RNA editing and organellar gene expression. Furthermore, 76 genes involved in biotic stress responses and plant defensins were suppressed, highlighting a potential compromise in innate immunity. Among the downregulated genes, 62 encode pectin methylesterase inhibitor (PMEI) proteins, which are crucial for regulating cell wall structure during reproduction. PMEIs inhibit the activity of pectin methylesterases (PMEs) by binding to them, thereby controlling the demethylesterification of homogalacturonan, a major component of pectin. This regulation is essential for the proper development of the intine layer, pollen germination, and pollen tube growth (Paynel et al., 2014; Zhang et al., 2010). A decrease in the expression of pectin methylesterase inhibitors (PMEIs) in siliques also may disrupt the balance of pectin modifications and potentially enhance pectin degradation and compromise cell adhesion during seed development (Okawa et al., 2023). Moreover, the significant downregulation of flavonoid biosynthesis genes, which influence pollen viability and anther dehiscence (Xu et al., 2015), provides an additional explanation for the observed fertility defects in the *galt* octuple mutants. The smaller siliques and reduced seed set observed the *galt* octuple mutant correlate with transcriptomic changes affecting suspensor development and early embryo formation. Downregulation of suspensor-specific genes, which are critical for nutrient transport to the developing embryo (Liu et al., 2015), likely contribute to compromised seed viability.

In addition, 52 genes associated with cell wall synthesis and modification were repressed, including expansins and structural glycoproteins, suggesting impaired cell wall remodeling. Genes encoding polygalacturonases (PGs) and pectate lyase-like proteins (PLLs) were also among those downregulated. PGs catalyze the hydrolysis of demethylesterified pectin by cleaving the links between the polymers that make up the cell wall, altering its extensibility and cell-cell adhesion (Kim et al., 2006). This process is important for pollen wall development, pollen tube growth and seed germination (Hadfield and Bennett, 1998; Sander et al., 2001). An alteration in the expression of these PGs can lead to a lack of coordination of cell wall loosening, which in turn affects associated developmental processes. Also, PLLs degrade pectin via β-elimination in a Ca^2+^-dependent manner. During pollen germination, PLLs play a key role in loosening the intine layer and facilitating pollen tube emergence (Chebli and Geitmann, 2018). These changes further support a role for AGP glycosylation in reproductive wall integrity. Indeed, future biochemical analysis of the changes in pectin in specific tissues would provide a better understanding of such AGP-pectin interactions. Also suppressed were 67 GDSL-motif lipase genes, many of which contribute to lipid remodeling and defense, 41 glutaredoxin-related redox genes, and 47 genes encoding pectin esterases, underscoring the widespread alterations in wall biogenesis, signaling, and redox homeostasis.

In contrast, the upregulated gene set reveals a notable shift toward signaling and defense-related transcriptional programs in both flowers and siliques, indicative of a compensatory response to compromised AGP glycosylation. Among these, 30 DUF26 domain-containing receptor-like kinases (RLKs) were significantly induced. DUF26-RLKs, also known as cysteine-rich receptor-like kinases (CRKs), are implicated in reactive oxygen species (ROS) sensing, immune activation, and stress adaptation (Wrzaczek et al., 2013). These proteins often function at the cell surface to perceive extracellular cues and relay stress or pathogen-associated signals to intracellular pathways. Their upregulation suggests heightened surveillance and potential compensatory reinforcement of defense signaling networks in the *galt* mutant. This suggests that the mutant is experiencing heightened stress, possibly as a compensatory mechanism for impaired development.

Also elevated were 16 wall-associated receptor kinases (WAKs). WAKs are pivotal in cell wall integrity sensing, acting as transducers that bind pectin fragments and initiate downstream responses related to growth, development, and pathogen resistance (Brutus et al., 2010; Kohorn and Kohorn, 2012). The increased expression of WAK genes aligns with the hypothesis that perturbation of AGP glycosylation compromises the mechanical or biochemical properties of the cell wall, thereby activating wall integrity surveillance pathways (Kohorn, 2016). Additionally, 9 Wheat LRK10-like RLKs (LRK10L-RLKs) were significantly upregulated. Originally characterized in wheat as genes involved in leaf rust resistance, LRK10L-RLKs in Arabidopsis and other dicots have been associated with broad-spectrum disease resistance, abiotic stress responses, and regulation of root and shoot development (Chern et al., 2001; Shiu and Bleecker, 2001). Their upregulation in *galt* octuple mutant flowers suggests a link between AGP function and broader receptor-mediated immunity and developmental robustness. Together, the induction of DUF26-RLKs, WAKs, and LRK10L-RLKs reflects an active reprogramming of the cell’s extracellular sensing and defense machinery. This pattern underscores the importance of AGPs not only as structural components of the wall matrix but also as modulators of receptor availability, ligand presentation, and cell surface signaling fidelity. Furthermore, we observed the induction of 46 glutathione transferase (GST) genes, 54 genes associated with redox ascorbate and glutathione pathways, and 52 peroxidase genes (**Supplementary Table 1**), indicates an attempt to mitigate oxidative damage and maintain cellular homeostasis (Gadjev et al., 2006), reflecting enhanced oxidative stress responses likely triggered by disruptions in cell wall and glycoprotein integrity. Strong upregulation of multiple receptor kinases and genes involved in signal transduction, redox homeostasis, and peroxidases, indicates activation of stress and wall integrity sensing pathways (Brutus et al., 2010; Chern et al., 2001; Kohorn and Kohorn, 2012; Wrzaczek et al., 2013).

### Cell wall organization and integrity

The gene set enrichment analysis of the *galt* mutant flowers show the downregulation of genes associated with cell wall organization, including key structural and regulatory components such as *EXPA25* and *EXPA16* (expansins) which are known to disrupt the non-covalent bonding between cellulose microfibrils and matrix glucans in order to allow cell wall loosening and extension. We also identified *PMEPCRF* which is a pectin methylesterase inhibitor, and *XTH19* (a xyloglucan endotransglucosylase/hydrolase). Xyloglucan endotransglucosylase/hydrolases (XTHs) facilitate the breakdown of xyloglucan polymers internally, while simultaneously incorporating new xyloglucan polysaccharides into the cell wall. This dual action helps in maintaining the cell wall’s thickness and structural integrity during cell elongation and organ growth (Cosgrove, 2018; Rose et al., 2002; Yokoyama and Nishitani, 2004). This widespread repression suggests that proper AGP glycosylation is essential for maintaining the transcriptional programs required for dynamic cell wall modulation, which is crucial to plant growth and development.

Gene ontology enrichment analysis of the commonly upregulated genes revealed a coordinated activation of stress-related biological processes, including responses to bacteria, external stimuli, and oxidative challenges, alongside the upregulation of molecular functions such as kinase activity, calcium ion binding, and phosphotransferase activity. These changes might indicate a shift toward a defense-primed physiological state, as plants often activate kinase-mediated signaling cascades and redox-responsive pathways in response to compromised cell wall integrity (Baez et al., 2022; Narváez-Barragán et al., 2022). The co-enrichment of carbohydrate- and polysaccharide-binding domains also suggests increased surveillance of cell wall status, consistent with previously reported compensatory signaling upon AGP deficiency (Hromadová et al., 2021).

AGPs modulate cell wall plasticity and architecture by interacting with pectins, hemicelluloses, and structural proteins, as well as by influencing signal transduction cascades (Seifert, 2021; Showalter and Basu, 2016). The observed suppression of wall-modifying genes likely reflects the absence of AGP-mediated signaling cues that normally promote wall loosening and extension, particularly in reproductive tissues where cell wall remodeling is critical. For instance, reduced expansin expression may contribute to compromised silique elongation and ovule penetration (Tan et al., 2024), phenotypes characteristic of the octuple mutant (Kaur et al., 2023; Moreira et al., 2024). Despite this general downregulation, of note is the upregulation of genes such as the FUT4 glycosyltransferase. FUT4 is responsible for adding terminal fucose residues to the galactan side chains of AGPs (Tryfona et al., 2012, 2014). In *galt* mutants, the initial galactosylation of hydroxyproline residues is severely impaired, resulting in truncated AG polysaccharide chains. As a result, substrates for FUT4 are largely missing or reduced. The observed upregulation of FUT4 may therefore represent a compensatory, rather than restorative transcriptional response to perceived defects in glycoprotein maturation or cell wall structure. The induction of AGP40, AGP5, LRX2, EXT3, and COBL5 may also reflect compensatory or stress-induced responses to wall perturbation. Proteins such as LRX2, a leucine-rich repeat extensin-like protein, play important roles in maintaining wall integrity and mechanosensing during root hair development (Borassi et al., 2020). Extensins like EXT3 contribute to wall strength and architecture (Lamport et al., 2011), while COBL5 participates in cellulose microfibril organization essential for anisotropic growth. These genes are typically activated in conditions of mechanical disturbance or wall perturbation, supporting the hypothesis that AGPs function not only as structural elements but also as sensors of cell wall integrity (Nguema-Ona et al., 2013). These findings position AGPs as central regulators of wall-associated transcriptional networks. Their glycosylation status appears to dictate the balance between wall extensibility and rigidity by shaping gene expression responses to developmental and environmental cues. They function in the loosening and remodeling of the cell wall for growth and development.

### The role of AGP glycosylation in plant development provides insight to the mechanism of action of AGPs

**Figure 7:**
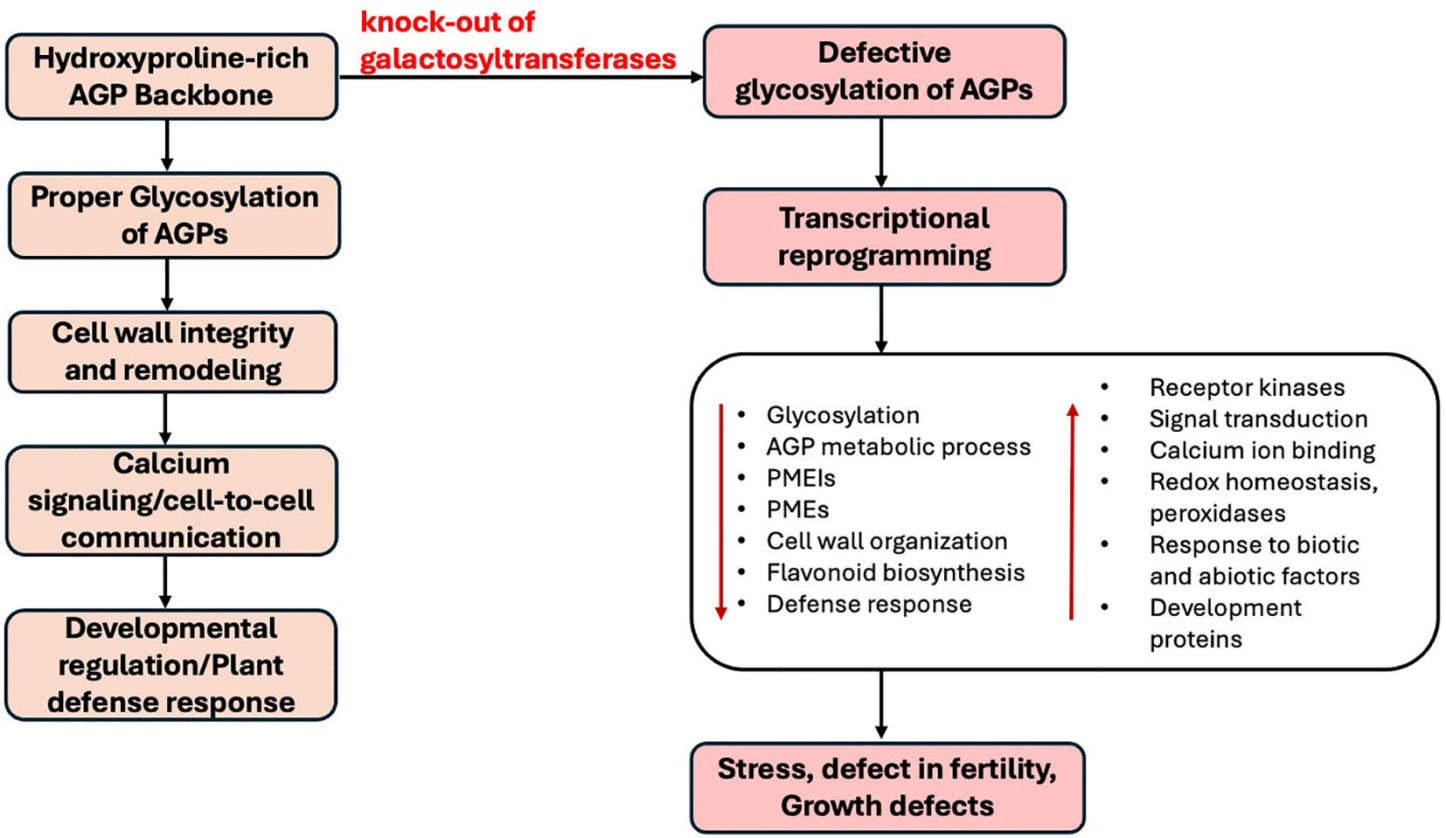
A pathway summary showing pathways associated with arabinogalactan proteins (AGPs) function. This figure demonstrates that proper glycosylation of AGPs is crucial for cell wall integrity and remodeling, leading to calcium signaling, cell-to-cell communication, developmental regulation, and plant defense responses. Conversely, a knock-out of galactosyltransferases results in defective glycosylation of AGPs, triggering transcriptional reprogramming that impacts various cellular processes, ultimately leading to stress, defects in fertility, and growth defects.

**Figure 7** summarizes the integrated transcriptomic response to AGP hypoglycosylation. Rather than a mechanistic model, it serves as a conceptual framework highlighting key pathways, glycosylation, signaling, and stress responses, altered in *galt* mutants. Proper AGP function begins with the hydroxyproline-rich protein backbone synthesized in the ER (Showalter, 2001; (Hijazi et al., 2014), followed by essential glycosylation catalyzed by galactosyltransferases (GALTs), mainly from the GT31 family. This large carbohydrate moiety provides the intermolecular surface for AGPs to maintain cell wall integrity and mediate calcium signaling and cell-to-cell communication at the cell surface. Properly glycosylated AGPs contribute directly to cell wall integrity and remodeling by interacting with pectin, cellulose, and other wall matrix components (Ellis et al., 2010; Tan et al., 2013). AGPs also serve as scaffolds that organize wall architecture and support wall extensibility, especially during reproductive development (Nguema-Ona et al., 2012; Pereira et al., 2016). These glycosylated AGPs are involved in calcium signaling and cell-to-cell communication, possibly acting as calcium capacitors that modulate cytosolic calcium levels, a key secondary messenger in plant development and stress signaling (Lamport et al., 2018; Lamport and Várnai, 2013). Their role at the cell surface, as well as their carbohydrate side chains allows them to integrate external signals (Lin et al., 2022) and regulate cellular processes through interactions with plasma membrane proteins, including receptor kinases. The loss of the eight *GALT* genesdisrupts this glycosylation, which triggers widespread transcriptional reprogramming. This defective glycosylation results in the significant downregulation of genes critical for growth and reproduction, including those for PMEIs, cell wall organization, and flavonoid biosynthesis. Simultaneously, the plant activates compensatory pathways by strongly upregulating components of stress and wall integrity sensing, particularly multiple receptor kinases. The ultimate downstream consequence of this disruption and compensatory signaling is stress, fertility defects, and growth impairments observed in the mutants (Kaur et al., 2023; Moreira et al., 2024).

This study strengthens the view that AGPs are integrative components of cell wall architecture and receptor-mediated signaling pathways. Their galactosylation is crucial for the initiation and proper glycosylation of AGPs, which in turn appears to be essential for proper gene regulation across multiple developmental programs. These findings provide a foundation for future functional studies on specific AGP-receptor interactions and highlights glycosylation as a critical layer of post-translational control in cell wall-mediated signaling and development. Overall, our findings collectively advance the understanding of the mechanism of action of AGPs by demonstrating their central role in coordinating developmental signaling, structural integrity, and stress responsiveness in reproductive tissues. AGPs are not merely structural glycoproteins but dynamic mediators of gene regulation, wall remodeling, and extracellular signal integration. Their glycosylation status directly impacts transcriptional networks essential for plant reproductive development and stress adaptation. To further uncover their mechanism of action, future studies should prioritize identifying specific AGP-interacting partners, such as receptor-like kinases, wall-modifying enzymes, and signaling molecules, to biochemically define the precise molecular interfaces through which AGPs influence cell fate, morphogenesis, and stress responses. Finally, while our transcriptomic data indicates strong associations between AGP glycosylation defects and altered pathways, these findings are largely correlative, and further studies with less severe mutants or additional time points may also help to elucidate direct interactions.

## Data Availability

The datasets presented in this study can be found in online repositories. The names of the repository/repositories and accession number(s) can be found below: https://www.ncbi.nlm.nih.gov/, PRJNA1338983.

## Supplementary material

Supplementary Figure 1 Functional enrichment analysis of shared and organ-specific differentially expressed genes in *galt* octuple mutant flowers and siliques was performed with respect to biological processes (B in red) and molecular functions (M in red). **(A)** Gene ontology (GO) enrichment analysis for the 12 genes downregulated in flowers but upregulated in siliques. **(B)** Enrichment analysis for the 7 genes upregulated in flower but downregulated in silique tissues. The size of the nodes indicates the number of genes contributing to that enrichment pathway from the dataset while the connecting lines indicate the co-interaction of the pathways. The darker the lines, the stronger the interactions. Node interaction was generated by ShinyGO v.0.86 (Ge et al., 2020).
