# Supplementary figures and images for: Transcriptomic insights into arabinogalactan protein mechanism of action in galactosyltransferase octuple mutants
Source: Front Plant Sci. 2026 Jan 16;16:1706954. doi: 10.3389/fpls.2025.1706954 (PMC12855564; doi:10.3389/fpls.2025.1706954)

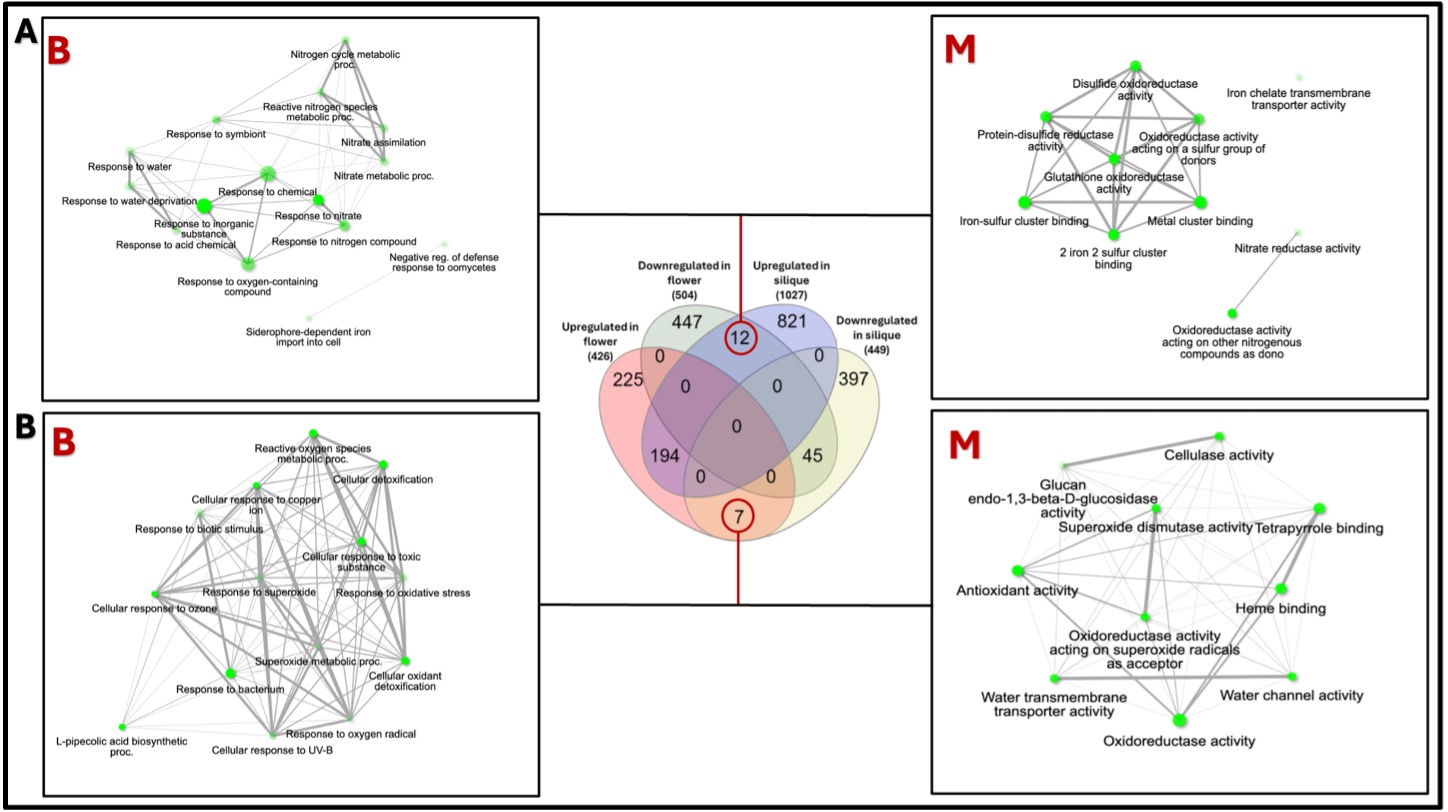

Supplement: Supplementary Figure 1 — Functional enrichment analysis of shared and organ-specific differentially expressed genes in galt octuple mutant flowers and siliques was performed with respect to biological processes (B in red) and molecular functions (M in red). (A) Gene ontology (GO) enrichment analysis for the 12 genes downregulated in flowers but upregulated in siliques. (B) Enrichment analysis for the 7 genes upregulated in flower but downregulated in silique tissues. The size of the nodes indicates the number of genes contributing to that enrichment pathway from the dataset while the connecting lines indicate the co-interaction of the pathways. The darker the lines, the stronger the interactions. Node interaction was generated by ShinyGO v.0.86 (Ge et al., 2020). [file Image1.jpeg]
