# Supplementary material for: Transcriptomic insights into arabinogalactan protein mechanism of action in galactosyltransferase octuple mutants
Source: Front Plant Sci. 2026 Jan 16;16:1706954. doi: 10.3389/fpls.2025.1706954 (PMC12855564; doi:10.3389/fpls.2025.1706954)
Supplement: Supplementary file 2 [file Table1.docx]

***Supplementary Table 1.*** Differential expression of peroxidase genes in galt octuple mutant flowers. This table shows both upregulated and downregulated peroxidase-related transcripts, with corresponding log2 fold change (log2FC) and adjusted p-values (padj).

| PEROXIDASES | | | | | | | |
| --- | --- | --- | --- | --- | --- | --- | --- |
| Gene_ID | Symbol | log2FC | padj | Gene_ID | Symbol | log2FC | padj |
| AT2G38380 | AT2G38380 | -0.93 | 0.423 | AT3G28200 | AT3G28200 | 0.13 | 0.705 |
| AT5G47000 | AT5G47000 | -0.73 | 0.450 | AT5G05340 | PRX52 | 0.15 | 0.766 |
| AT1G24110 | AT1G24110 | -0.67 | 0.570 | AT4G21960 | PRXR1 | 0.31 | 0.127 |
| AT2G37130 | AT2G37130 | -0.48 | 0.618 | AT5G15180 | AT5G15180 | 0.35 | 0.497 |
| AT4G33870 | AT4G33870 | -0.46 | 0.118 | AT5G58390 | AT5G58390 | 0.41 | 0.276 |
| AT2G34060 | AT2G34060 | -0.45 | 0.264 | AT5G06720 | PA2 | 0.41 | 0.166 |
| AT5G64110 | AT5G64110 | -0.41 | 0.773 | AT4G37520 | AT4G37520 | 0.41 | 0.493 |
| AT4G17690 | AT4G17690 | -0.36 | 0.533 | AT3G21770 | AT3G21770 | 0.69 | 0.032 |
| AT1G44970 | AT1G44970 | -0.35 | 0.0008 | AT5G19890 | AT5G19890 | 0.72 | 0.357 |
| AT4G16270 | AT4G16270 | -0.28 | 0.353 | AT4G36430 | AT4G36430 | 0.74 | 0.255 |
| AT1G71695 | AT1G71695 | -0.22 | 0.0031 | AT3G49110 | PRXCA | 0.90 | 0.160 |
| AT5G58400 | AT5G58400 | -0.17 | 0.761 | AT2G18150 | AT2G18150 | 1.00 | 0.00033 |
| AT3G32980 | AT3G32980 | -0.14 | 0.832 | AT3G03670 | AT3G03670 | 1.05 | 0.0081 |
| AT4G30170 | AT4G30170 | -0.11 | 0.920 | AT4G11290 | AT4G11290 | 1.10 | 0.00018 |
| AT5G51890 | AT5G51890 | -0.10 | 0.672 | AT4G37530 | AT4G37530 | 1.22 | 0.00042 |
| AT5G40150 | AT5G40150 | -0.06 | 0.852 | AT5G42180 | PER64 | 1.41 | 2.39E-09 |
| AT2G22420 | AT2G22420 | -0.02 | 0.957 | AT5G06730 | AT5G06730 | 1.43 | 0.0040 |
| AT5G64120 | AT5G64120 | -0.01 | 0.994 | AT2G41480 | AT2G41480 | 1.45 | 0.000424 |
| AT4G25980 | AT4G25980 | 0.04 | 0.973 | AT4G08780 | AT4G08780 | 1.56 | 0.104 |
| AT5G66390 | AT5G66390 | 0.08 | 0.894 | AT5G19880 | AT5G19880 | 1.68 | 4.20E-06 |

***Supplementary Table 2***. Differential expression of storage protein and late embryogenesis abundant (LEA) protein genes in galt octuple mutant flowers. This table presents genes associated with developmental processes, showing both upregulated and downregulated transcripts with corresponding log2 fold change (log2FC) and adjusted p-values (padj).

| DEVELOPMENT. STORAGE PROTEINS | | | | DEVELOPMENT. LATE EMBRYOGENESIS ABUNDANT | | | |
| --- | --- | --- | --- | --- | --- | --- | --- |
| Symbol | ID | log2FC | padj | Symbol | GeneID | log2FC | padj |
| AT1G03880 | AT1G03880 | 3.89 | 0.10 | AT2G44060 | AT2G44060 | -0.18 | 0.21 |
| AT1G03890 | AT1G03890 | 5.04 | 0.09 | AT2G46140 | AT2G46140 | -0.75 | 0.0014 |
| AT1G07750 | AT1G07750 | -0.43 | 9.79E-05 | AT3G15670 | AT3G15670 | 5.98 | 0.0004 |
| AT1G33270 | AT1G33270 | 0.26 | 0.81 | AT3G53040 | AT3G53040 | 2.15 | 0.03 |
| AT2G26560 | AT2G26560 | 3.61 | 0.01 | AtLEA4-1 | AT1G32560 | 2.09 | 0.04 |
| AT2G28490 | AT2G28490 | 2.50 | 0.31 | LEA14 | AT1G01470 | 2.55 | 6.52E-06 |
| AT2G28680 | AT2G28680 | -0.58 | 0.16 | LEA18 | AT2G35300 | 0.75 | 0.46 |
| AT2G39220 | AT2G39220 | 0.04 | 0.93 | LEA3 | AT1G02820 | 1.50 | 0.11 |
| AT3G22640 | AT3G22640 | 2.33 | 0.39 | LEA4-5 | AT5G06760 | 3.14 | 3.24E-08 |
| AT3G54950 | AT3G54950 | 2.44 | 7.57E-05 | SAG21 | AT4G02380 | 3.14 | 0.02 |
| AT3G63200 | AT3G63200 | -0.41 | 0.18 | AT1G72100 | AT1G72100 | 3.23 | 0.007 |
| AT4G27140 | AT4G27140 | 4.91 | 0.008 | AT2G03850 | AT2G03850 | 2.54 | 0.01 |
| AT4G27150 | AT4G27150 | 3.34 | 0.15 | AT3G17520 | AT3G17520 | 4.02 | 2.38E-08 |
| AT4G27160 | AT4G27160 | 3.16 | 0.20 | AT4G21020 | AT4G21020 | 2.46 | 0.02 |
| AT4G27170 | AT4G27170 | 3.341 | 0.17 | AT5G44310 | AT5G44310 | 1.78 | 0.64 |
| AT4G37050 | AT4G37050 | 0.39 | 0.64 |  |  |  |  |
| AT5G04040 | AT5G04040 | 0.41 | 0.15 |  |  |  |  |
| AT5G19120 | AT5G19120 | 0.30 | 0.71 |  |  |  |  |
| AT5G24770 | AT5G24770 | 0.90 | 0.003 |  |  |  |  |
| AT5G24780 | AT5G24780 | 0.13 | 0.84 |  |  |  |  |
| AT5G44020 | AT5G44020 | -0.65 | 0.08 |  |  |  |  |
| AT5G44120 | AT5G44120 | 2.57 | 0.41 |  |  |  |  |
| AT5G44120 | AT5G44120 | 2.40 | 0.46 |  |  |  |  |
| AT5G44120 | AT5G44120 | 2.16 | 0.43 |  |  |  |  |

***Supplementary Table 3.* Differential expression of NAC domain transcription factor genes in** galt **octuple mutant flowers. This table lists both upregulated and downregulated NAC transcription factors, with corresponding log2 fold change (log2FC) and adjusted p-values (padj).**

| TRANSCRIPTION FACTOR FAMILY NAC DOMAIN | | | |
| --- | --- | --- | --- |
| Symbol | DEG | log2FC | padj |
| NAC044 | AT3G01600 | -0.64 | 0.22 |
| NAC045 | AT3G03200 | -0.52 | 0.57 |
| CUC1 | AT3G15170 | -0.35 | 0.57 |
| NAC1 | AT1G56010 | 0.29 | 0.57 |
| TIP | AT5G24590 | 0.48 | 0.11 |
| NAC2 | AT3G15510 | 0.49 | 0.26 |
| NAC041 | AT2G33480 | 0.59 | 0.36 |
| NAC028 | AT1G65910 | 0.67 | 0.18 |
| NAC074 | AT4G28530 | 2.35 | 3.70E-05 |
| AT3G12910 | AT3G12910 | 3.65 | 0.02 |
| NAC3 | AT3G15500 | 4.27 | 2.55E-07 |
| NAC047 | AT3G04070 | 5.16 | 1.68E-11 |
| NAC061 | AT3G44350 | 5.33 | 0.011 |
