# Supplementary material for: Transcriptomic insights into arabinogalactan protein mechanism of action in galactosyltransferase octuple mutants
Source: Front Plant Sci. 2026 Jan 16;16:1706954. doi: 10.3389/fpls.2025.1706954 (PMC12855564; doi:10.3389/fpls.2025.1706954)
Supplement: Supplementary file 3 [file Table2.docx]

***Supplementary table 4*. The regulation of enzymes vital to AGP glycosylation (Glycoside hydroxylases) in flowers and siliques.**

| GLYCOSIDE HYDROLASE GENE FAMILIES (FLOWERS) | | | | GLYCOSIDE HYDROLASE GENE FAMILIES (SILIQUES) | | | |
| --- | --- | --- | --- | --- | --- | --- | --- |
| Gene_ID | Symbol | log2FC | padj | Gene_ID | Symbol | log2FC | padj |
| AT1G05850 | POM1 | 0.32 | 0.78 | AT1G05850 | POM1 | -0.29 | 0.71 |
| AT3G16920 | CTL2 | 0.09 | 0.84 | AT3G16920 | CTL2 | 0.46 | 0.07 |
| AT3G47540 | AT3G47540 | 0.75 | 0.02 | AT3G47540 | AT3G47540 | 0.72 | 0.30 |
| AT3G54420 | EP3 | 0.37 | 0.35 | AT3G54420 | EP3 | 1.08 | 0.08 |
| AT3G12500 | HCHIB | 0.48 | 0.23 | AT3G12500 | HCHIB | 1.12 | 0.004 |
| AT4G01700 | AT4G01700 | 1.20 | 0.0002 | AT4G01700 | AT4G01700 | 1.27 | 0.012 |
| AT2G43620 | AT2G43620 | -0.02 | 0.99 | AT2G43620 | AT2G43620 | 2.09 | 0.0067 |
| AT2G43590 | AT2G43590 | -0.29 | 0.76 | AT2G43590 | AT2G43590 | 2.25 | 0.0031 |
| AT2G43580 | AT2G43580 | 2.65 | 0.0069 | AT2G43580 | AT2G43580 | 4.57 | 0.00054 |
| AT2G43570 | CHI | 4.11 | 8.60E-39 | AT2G43570 | CHI | 4.63 | 2.78E-05 |
